# Heme Metabolism‐Derived Carbon Monoxide Regulates Skeletal Muscle Function

**DOI:** 10.1002/jcsm.70309

**Published:** 2026-05-14

**Authors:** Rodrigo W. Alves de Souza, Hyo In Kim, Paula Ketilly Nascimento Alves, Ailma Oliveira da Paixão, Ashlee Rasmussen, Sidharth Shankar, James Harbison, Vanessa Azevedo Voltarelli, Leo E. Otterbein

**Affiliations:** ^1^ Department of Surgery, Beth Israel Deaconess Medical Center Harvard Medical School Boston Massachusetts USA; ^2^ School of Physical Education and Sport University of São Paulo SP Brazil; ^3^ Department of Pharmacology, College of Korean Medicine Kyung Hee University Seoul Republic of Korea

**Keywords:** carbon monoxide, exercise adaptation, heme oxygenase, mitochondrial dysfunction, neuromuscular junction, skeletal muscle metabolism

## Abstract

**Background:**

Heme oxygenases, HO‐1 (*Hmox1*) and HO‐2 (*Hmox2*), regulate skeletal muscle homeostasis by degrading heme and generating carbon monoxide (CO), a bioactive signalling molecule. Although HO‐1 is known to influence muscle fibre composition and mitochondrial function, the role of HO‐2 in activity‐dependent neuromuscular plasticity remains poorly understood. This study aimed to define the distinct contributions of each isoform and test whether CO could restore muscle function in HO‐deficient states.

**Methods:**

We generated Hmox1/2 double‐knockout mice (*Hmox1/2* ^*−/−*^) and compared their skeletal muscle phenotype with that of single HO‐1 or HO‐2 knockouts and wild‐type (WT) controls under sedentary and exercised conditions. We evaluated endurance capacity using treadmill running (*n* = 8–12 per group), assessed fibre‐type distribution and neuromuscular junction (NMJ) morphology via immunohistochemistry and measured mitochondrial function using high‐resolution respirometry. Primary neuronal cultures were analysed using multielectrode array recordings to assess firing dynamics. Inhaled CO was administered to test its capacity to rescue muscle phenotype and performance.

**Results:**

HO‐1 deficiency led to a significant reduction in oxidative fibres (Type I and IIa), decreased mitochondrial respiratory capacity (reduced by ~30%, *p* < 0.01) and diminished treadmill endurance (−40% running time vs. WT, *p* < 0.001). Hmox2 deficiency was associated with NMJ remodelling, increased acetylcholine receptor expression, reduced Sox2 transcription and heightened burst firing. The double deletion of HO‐1/HO‐2 produced an additive phenotype characterized by severe mitochondrial dysfunction, increased glycolytic fibre content and NMJ remodelling. We identify CO, a by‐product of HO‐1, as a crucial modulator of skeletal muscle adaptation, capable of compensating for HO deficiency. Treatment with CO in *Hmox1/2* ^*−/−*^ mice restored fibre‐type distribution toward oxidative fibres (increased by 25%, *p* < 0.01), improved mitochondrial respiratory parameters and doubled endurance performance (*p* < 0.001). CO also normalized mitochondrial protein expression and modulated key metabolic pathways, including nucleotide metabolism, the TCA cycle and redox balance.

**Conclusions:**

HO‐1 and HO‐2 have distinct roles in regulating muscle phenotype and metabolic adaptation. HO‐1 modulates mitochondrial content and muscle plasticity, whereas Hmox2 regulates, in part, activity‐dependent neuromuscular plasticity and responsiveness to exercise. Exogenous CO effectively restores mitochondrial and functional deficits in HO‐deficient muscle, mimicking endurance exercise adaptations. These findings support the therapeutic potential of CO in conditions of muscle disuse, aging or disease where exercise is limited or not feasible.

## 1 Introduction

Over the past 20 years, numerous studies have investigated CO as a gaseous mediator that elicits specific cellular responses across many cell types. This has resulted in CO being accepted as a bioactive gas, leading to its evaluation as a therapeutic at low doses [1]. The most intuitive method of CO administration is via inhalation, where the gas rapidly diffuses across the alveolar‐capillary interface and rapidly binds to haemoglobin [2]. Several clinical studies with inhaled CO predictably and reliably showed that CO can be administered safely at low doses in both healthy volunteers and those with respiratory complications [3, 4]. A study of healthy subjects treated with 100–200 ppm (0.01%) inhaled CO for 1 h for five consecutive days achieved a maximum carboxyhemoglobin (COHb) concentration of 3.3% with no adverse events [5]. Moreover, these CO‐treated subjects showed increased expression of protective genes in skeletal muscle biopsies and increased expression of mitochondrial fusion proteins in response to CO [4]. At physiologically safe amounts, CO can reduce the production of proinflammatory cytokines and increase the release of anti‐inflammatory molecules. Moreover, CO can protect tissues from oxidative stress by increasing antioxidant defence mechanisms and reducing the levels of reactive oxygen species [6]. In addition, CO increases mitochondrial biogenesis and capillary density in skeletal muscle [5].

Endogenously, CO is produced by the heme oxygenase (HO) enzymes HO‐1 (*Hmox1*) and HO‐2 (*Hmox2*) during heme degradation [1]. Although both HO enzymes utilize heme as a substrate, they differ in their location and associated functionality. As with other gene isoforms, they can compensate when either is absent [7]. HO‐2 has traditionally been considered a constitutively expressed isoform, found primarily in neurons and testes [8]. At the same time, HO‐1 is the inducible isoform and is expressed at low levels in skeletal muscle under basal conditions but becomes significantly upregulated in response to exercise training [9]. We have previously reported that mice lacking HO‐1 selectively in skeletal muscle fibres exhibit dramatic structural and functional remodelling, resulting in a faster and more fatigue‐prone phenotype. Further, the absence of HO‐1 within the muscle fibre partially impairs the benefits of aerobic exercise training by altering muscle phenotype within weeks after deletion [9]. This reflects changes in cellular metabolism and mitochondrial bioenergetics, suggesting that HO‐1 plays a critical role in both muscle plasticity and energy homeostasis.

Despite the well‐established role of HO‐1 in exercise adaptation and skeletal muscle health, the contribution of HO‐2 in skeletal muscle, particularly its contribution to neuromuscular regulation, remains poorly defined. To date, no studies have examined the impact of simultaneous genetic deletion of both isoforms on skeletal muscle physiology or investigated whether CO, as a product of HO activity, could compensate for the lack of HO. Here, we explore the combined genetic deletion of HO‐1 and HO‐2 to dissect their complementary roles in skeletal muscle development and function. We further examine whether exogenous CO supplementation can restore the physiological and metabolic deficits resulting from HO loss. Understanding these regulatory pathways may have important implications for conditions such as ischemia–reperfusion injury [10] and Duchenne muscular dystrophy [11], where muscle dysfunction and metabolic dysregulation are key pathological features. Our findings reveal new insights into HO/CO‐mediated regulation of muscle health and identify CO as a promising therapeutic agent capable of mimicking exercise‐induced adaptations.

## 2 Methods

### 2.1 Cell Culture and Experimental Animals

For muscle cell lines, mouse muscle myoblasts (C2C12; ATCC CRL‐1772) were transfected (RNAiMAx) with siRNA duplexes targeting HO‐1 (s67607) or scrambled control siRNA (100 pmol, Thermo Fisher Scientific) and continuously exposed to 250 ppm CO plus 5% CO_2_ for 72 h. Primary neurons were isolated from the cortex and hippocampus of E16 C57BL6 (WT) and constitutive HO‐2 knockout (*Hmox2* ^*−/−*^) embryos using an adapted papain‐based dissociation protocol [12]. Cells were cultured on 24‐well MEA plates. Extracellular activity was recorded at baseline (0 h), 24 h, and 48 h for 5 min each. Signals were analysed with AxIS Navigator and AxIS Metric Plotting Tools. Primary outcomes were Weighted Mean Firing Rate (Hz) and Burst Percentage (%), with parameters consistent across genotypes and time points. At least three independent preparations were analysed.

For in vivo animal studies, male and female WT mice were purchased from Charles River or Jackson Laboratory (25–30 g). Constitutive *Hmox2* ^*−/−*^ mice were kindly provided by Sylvain Doré (University of Florida, Gainesville, FL). Global HO‐1 knockout mice (*Rosa26; R26‐Hmox1* ^*−/−*^) were generated by breeding *Hmox1* ^*fl/fl*^ mice with mice expressing Cre recombinase under the oestrogen receptor T2 (Jackson Laboratory, Stock# 008463). To generate HO‐1 and HO‐2 double‐knockout mice, *R26‐Hmox1* ^*−/−*^ strain were crossed with *Hmox2* ^*−/−*^ animals, creating *R26‐Hmox1/2* ^*−/−*^. Upon the tamoxifen regimen, both *R26‐Hmox1* ^*−/−*^ and *R26‐Hmox1/2* ^*−/−*^ (*Hmox1/2* ^*−/−*^) strains exhibited deletion of single HO‐1 and double HO‐1/2. Age‐matched controls consisted of *Hmox1* ^*fl/fl*^ and olive oil vehicle (*olive oil‐Rosa26‐Cre‐Hmox1‐Hmox2* ^*+/−*^), referred to as *Hmox1/2* ^*+/−control*^, which has a deletion of HO‐2 and normal expression levels of HO‐1. Tamoxifen‐induced skeletal muscle‐specific HO‐1 knockout mice (*MHmox1* ^*−/−*^) were generated as previously described [9]. The diaphragm, tibialis anterior (TA), soleus, gastrocnemius and plantaris muscles were carefully harvested and stored at −80°C. An expanded methods section is provided in the Supporting Information section.

### 2.2 Low‐Dose Inhaled CO Model

Treatment with CO involves exposure to CO or medical‐grade air. CO was administered either as a short‐term exposure for 14 consecutive days or as a long‐term intermittent treatment concomitant with aerobic exercise training. CO was administered to mice for 1 h/day in a custom‐made gas exposure chamber where mice had free access to food and water. CO gas (1%) was mixed with air to achieve 250 ppm; (0.025%). An infrared gas analyser (Interscan) monitored the CO concentration continuously throughout the application. For the 6‐week aerobic exercise training protocol, animals were exposed to CO or synthetic air for 1 h before each exercise training session. To evaluate the haematological parameters, such as haematocrit and COHb levels, mice were exposed to 14 days of CO, as described above. Within 5 min after the last exposure, blood was collected under anaesthesia. All blood was placed into 1‐ml BD syringes filled with 100 U of heparin and analysed on a Radiometer ABL80 FLEX analyser. For muscle CO levels, male mice were exposed to CO (250 ppm) for 1 h and then immediately removed to evaluate the concentration of CO in the soleus, diaphragm, tibialis anterior and plantaris muscles. The concentration of CO in muscle tissue was measured as previously described [13].

### 2.3 Treadmill Exercise and Aerobic Training Protocols

The acute running capacity was evaluated by a graded exercise test on a treadmill (Exer 3/6, Columbus Instruments, USA), which started at 6 m/min, with the speed increasing by 3 m/min every 3 min until the mice could not run due to exhaustion, as previously reported [9], and detailed in the Supporting Information section. Total distance run (metres) and peak workload (metres/min) were recorded. For the running training protocol, mice underwent moderate‐intensity aerobic exercise for 60 min, 5 days a week, for 6 weeks at 60% of the maximal workload achieved in the graded treadmill running test described above [9]. After 6 weeks of the training protocol, the samples were carefully harvested 72 h after the last exercise session.

### 2.4 In Vivo Metabolism Assessment and Mitochondrial Activity

Metabolic parameters and activity were assessed with a Columbus Instruments Comprehensive Lab Animal Monitoring System (CLAMS; Columbus Instruments, Columbus, OH) equipped with subsystems for open‐circuit indirect calorimetry and activity monitoring. All mice were acclimated to the monitoring cages for 24 h before an additional 48 h of hourly automated recordings of physiological parameters. Skeletal muscle mitochondria isolation and function were assessed as previously described [9]. All the procedures followed the manufacturer's instructions (Agilent Seahorse, XF Cell Mito Stress kit).

### 2.5 Histology

Fibre frequency and cross‐sectional area (CSA) were measured in the TA muscles after dissection, embedding in OCT, and flash‐frozen in liquid nitrogen. Immunofluorescence was performed as previously described [9]. Histological analysis was measured in whole muscle preparations at 10X magnification, and images were further analysed using the software ImageJ and SMASH‐Semiautomatic Image Processing of Skeletal Muscle Histology [14], respectively. Extensor digitorum longus (EDL) muscles were fixed in 4% PFA, cryoprotected in 30% sucrose and sectioned longitudinally at 30 μm. Sections were blocked (M.O.M., 1% Triton X‐100, 4% BSA) and incubated overnight at 4°C with antineurofilament (2H3), antisynaptic vesicle (SV2) and *α*‐Bungarotoxin‐ATTO‐488 to label pre‐ and postsynaptic NMJ components. After washing, sections were incubated with Alexa Fluor 594‐conjugated secondary antibody and mounted in VectaShield. Imaging was performed using an Olympus BX62 with a 60x objective and Andor Sona sCMOS camera. NMJ morphology was quantified in ImageJ using BinaryConnectivity and the NMJ‐morph protocol [15] by a blinded observer across ≥ 4 animals per genotype, detailed in the Supporting Information section.

### 2.6 RNA Reverse Transcription and qPCR (RT‐qPCR)

Total RNA was extracted from heart ventricles using TRIzol reagent (Invitrogen) following the manufacturer's instructions. Reverse transcription was performed using a High‐Capacity cDNA synthesis kit (Thermo Scientific). After cDNA synthesis, RT‐qPCR for target and endogenous genes (Table S1) ran separately, and amplifications were detected using QuantStudio3 (Applied Biosystems, Foster City, CA, USA) using Maxima SYBR Green/ROX qPCR Master Mix (Thermo Scientific). Fold‐change was calculated using the ΔΔCt method.

### 2.7 Immunoblotting

Protein extraction, quantification, preparation and immunoblotting experiments were performed as previously described [9]. Mitochondrial oxidative phosphorylation complexes (OXPHOS, ab110413), Hmox1 (ab52947) and Gapdh (ab9485) primary antibodies were purchased from Abcam. Mitofusin 1 (#14739), Mitofusin 2 (#9482), Opa1 (#80471) and Drp1 (#5391) were purchased from Cell Signalling. Secondary specific antibodies were goat antimouse IR800 and goat antirabbit IR680 and IR800 (LICOR). Membranes were scanned using the Odyssey Infrared Imaging System (LICOR).

### 2.8 Metabolite Measurement by LC/MS–MS

Muscle metabolite extraction and preparation were performed as previously described [16] and are detailed in the Supporting Information section. Peak areas from the total ion current for each metabolite were integrated using MultiQuant v.3.0 (AB/SCIEX). Metabolite total ion counts for a given transition were normalized to the protein content of matched lysates for each treatment group. Treatment replicates were then scaled around their replicate group means to normalize for run order effects between replicate groups. The resultant peak areas were subjected to relative quantitation analyses. Furthermore, statistical enrichment and pathway impact analyses were performed using MetaboAnalyst 6.0 software.

### 2.9 Statistical Analysis

The statistical analysis was performed using GraphPad Prism version 10 (GraphPad Software, La Jolla, CA). The Shapiro–Wilk normality test was used to assess the distribution of the data. Statistical data were tested for normal distribution and homogeneity. An unpaired 2‐tailed Student's *t*‐test was performed to compare the two groups. A two‐way ANOVA with repeated measures was used to analyse the effects of experimental factors at different time points. For the comparison among three or more groups, 1‐way ANOVA with Tukey's post hoc test was performed. No statistical methods were used to predetermine the sample size. All results are depicted as mean ± SD, and *p*‐values < 0.05 were considered significant.

## 3 Results

### 3.1 Lack of HO‐2 Interferes With Exercise Training Adaptation and Neuromuscular Morphology

**FIGURE 1:**
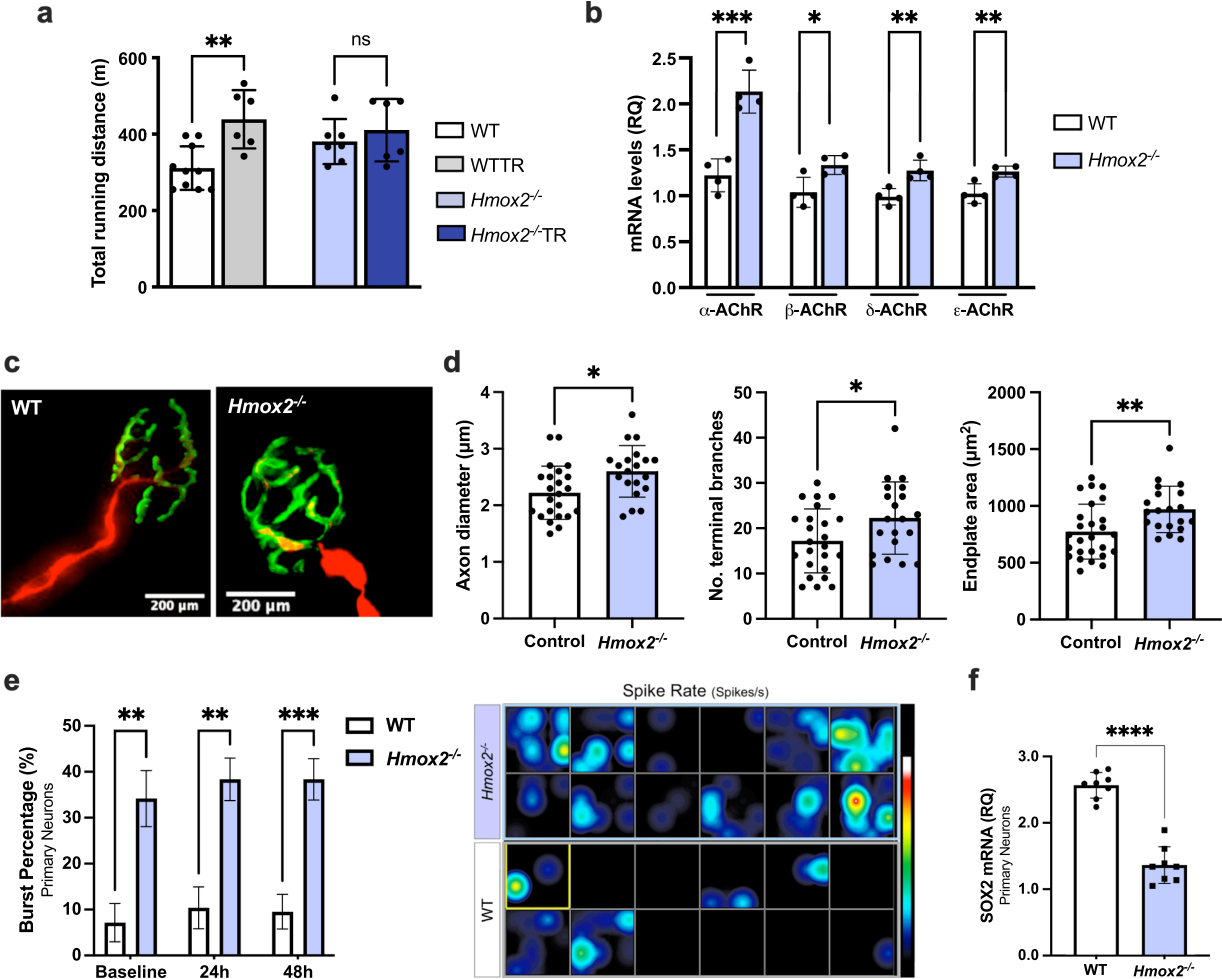
HO‐2 deficiency impairs exercise training adaptations and leads to NMJ remodelling. (a) Maximal treadmill running distance in control and *Hmox2* ^*−/−*^ groups, either submitted to or not to 6 weeks of running aerobic training (TR). (b) mRNA expression levels of acetylcholine receptor (AChRs) subunits in the plantaris muscle of WT and *Hmox2* ^*−/−*^ animals, normalized to Hprt1. *n* = 4–10 mice per group. (c) Representative immunofluorescent staining of extensor digitorum longus muscles from WT and *Hmox2* ^*−/−*^ mice, stained with anti‐*α*‐bungarotoxin (green) and antineurofilament, antisynaptic vesicle antibodies (red). (d) Mean axon diameter, number of terminal branches and endplate area were determined using ImageJ software along with the Binary Connectivity plugin. Each bar represents mean ± SD from a minimum of 20 NMJ from four mice/group. (e) Quantification of burst percentage (%) in WT and *Hmox2* ^*−/−*^ neuronal networks recorded at baseline (0 h), 24 and 48 h (left), and a representative spatial heat map of spike rate (spikes/s) across an entire 24‐well MEA plate. Each square corresponds to one well, and colour intensity indicates the mean spike rate and activity across active electrodes within that well. (f) Sox2 mRNA levels in primary neurons from the cortex and hippocampus of embryonic day E16 C57BL6 (WT) and *Hmox2* ^*−/−*^ mice. Data are presented as mean ± SD of three independent assays. *p < 0.05, **p < 0.01, ***p < 0.001 and ****p < 0.0001.

HO‐2, the constitutive isoform of heme oxygenase, was localized to the neuromuscular junction by Kusner et al., but its role remains unclear, with CO suggested as a potential neurotransmitter [17]. To study this, we examined HO‐2's contribution to skeletal muscle function in wild‐type (WT) and *Hmox2* ^*−/−*^ mice, which have about 80% less HO‐2 mRNA (Figure S1a). Notably, the absence of HO‐2 did not lead to increased HO‐1 expression in skeletal muscle (Figure S1a). Additionally, sedentary mice without HO‐2 showed no differences in running capacity, fibre‐type distribution or CSA compared to sedentary WT controls (Figure S1c). Satellite cell Muscle Regulatory Factors (MRFs) also influence fibre‐type transition and muscle mass, so we measured MyoD and myogenin as proliferation and differentiation markers, respectively [18]. The lack of morphological changes in these animals was further supported by unchanged MyoD and myogenin levels (Figure S1d). These findings suggest that HO‐2 is not crucial for baseline skeletal muscle structure and function. Regarding heme metabolism, trained HO‐1‐deficient mice (*R26‐Hmox1* ^*−/−*^) or *Hmox2* ^*−/−*^ mice exhibited a significant increase in circulating free heme after chronic aerobic training compared to WT, indicating that deficiency of either HO isoform compromises heme metabolism (Figure S1e). Moreover, *Hmox2* ^*−/−*^ mice showed significantly higher circulating heme levels compared to WT and *R26‐Hmox1* ^*−/−*^ mice, both before and after training, indicating a role for HO‐2 in maintaining heme homeostasis (Figure S1e), which we posited may impact neuromuscular signalling and exercise adaptation. We further investigated whether HO‐2 is vital for adaptive responses to exercise training. Notably, *Hmox2* ^*−/−*^ mice failed to improve their running capacity after 6 weeks of aerobic training, as shown by reduced fatigue time, unlike control mice under the same exercise regimen (Figure 1a).

Aerobic training adaptations occur at the NMJ, where motor neurons and skeletal muscle fibres meet [19]. This communication induces functional and structural changes in response to exercise, leading to enhanced neurotransmitter release, increased acetylcholine receptor density and improved synaptic transmission efficiency [19]. These adaptations enhance endurance by sustaining motor unit engagement and lowering neuromuscular fatigue [19]. Given the relevance of HO‐2 to the nervous system and its higher presence in the NMJ sarcolemma, we investigated its role in NMJ organization [17, 20]. We observed increased expression of the four skeletal acetylcholine receptor (AChRs) subunits in EDL from *Hmox2* ^*−/−*^ mice (Figure 1b). Pretzel‐shaped AChR aggregates in the EDL postsynaptic membrane were stained with AF488‐*α*‐bungarotoxin, whereas motor neurons were stained with antineurofilament and antisynaptic vesicle antibodies (Figure 1c). Quantification of pre‐ and postsynaptic parameters revealed that EDL neurons from *Hmox2* ^*−/−*^ mice had significantly larger axon diameters, a greater number of terminal branches and larger endplate areas as compared to those analysed from WT littermates (Figure 1d). Given the prominent neuronal localization of HO‐2 and the marked NMJ remodelling observed in *Hmox2* ^*−/−*^ mice, we next examined whether lack of HO‐2 had an impact on intrinsic neuronal properties that might contribute to neuromuscular plasticity. To assess functional consequences at the network level, neuronal activity was recorded using a microelectrode array (MEA) plate using primary neurons isolated from *Hmox2* ^*−/−*^ and WT mice. *Hmox2* ^*−/−*^ neurons exhibited a significantly higher neuron burst percentage basally that persisted over time (Figure 1e; Figure S1f). This correlated with a significant reduction in sex determining region Y‐box 2 (Sox2) mRNA levels (Figure 1f), a transcription factor regulated by activity‐dependent calcium signalling and involved in neuronal plasticity and maturation [21, 22]. These data indicate altered regulation of firing rather than reduced excitability. Collectively, these results underscore the important role of HO‐2 in organization of skeletal muscle tissue and neuronal synaptic morphology and function.

### 3.2 Absence of HO‐1 and HO‐2 Result in Muscle and NMJ Abnormalities

**FIGURE 2:**
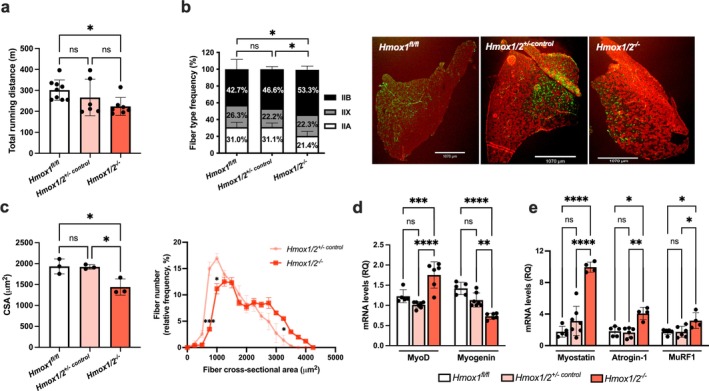
The absence of HO‐1 and HO‐2 promotes atrophy and susceptibility to fatigue. (a) Maximal running distance during the treadmill exhaustive test. (b) Fibre‐type frequency measurement and representative immunostaining images. Green, Type IIa MyHC isoform; red, Type IIb MyHC isoform; black, Type IIx MyHC isoform; turquoise, laminin. Scale bars for both groups: 1070 μm. *p < 0.05; *n* = 3 mice per group and five fields at 10x/muscle. (c) Quantitative morphometric evaluation of tibialis anterior muscles harvested from *Hmox1* ^*fl/fl*^, *Hmox1/2* ^*+/− control*^ and *Hmox1/2* ^*−/−*^ mice by mean fibre cross‐sectional area (CSA) and fibre size distribution. *p < 0.05, ***p < 0.001 versus *Hmox1/2* ^*+/− control*^; *n* = 3/group and five fields at 10x/muscle. (d) mRNA expression of plantaris muscle MRFs and (e) proteolytic system components in *Hmox1* ^*fl/fl*^, *Hmox1/2* ^*+/− control*^ and *Hmox1/2* ^*−/−*^ mice. Data represent mean ± SD of 4–7 mice/group. *p < 0.05, **p < 0.01, ***p < 0.001 and ****p < 0.0001.

Given the roles of HO‐1 and HO‐2 in skeletal muscle, we generated double‐knockout mice (*Hmox1/2* ^*−/−*^) by breeding *R26‐Hmox1* ^*−/−*^ littermates with *Hmox2* ^*−/−*^. This facilitated HO‐1 knockdown through tamoxifen administration (Figure S2a), allowing us to study effects beyond myocytes in adult animals. *Hmox1/2* ^*−/−*^ mice exhibited a 60% reduction in HO‐1 and a 50% decrease in HO‐2 levels posttamoxifen compared to age‐matched controls (Figure S2b,c). Although we previously described HO‐1‐deficient skeletal muscle (*MHmox1* ^*−/−*^), the global deletion of HO‐1 also demonstrated similar muscle abnormalities (Figure S2d,e). To assess the impact on muscle function, we evaluated running capacity, which was 36% less in *Hmox1/2* ^*−/−*^ mice compared to controls (301 vs. 223 min, *p* < 0.05; Figure 2a). We investigated tibialis anterior muscle morphology and found significant alterations in fibre‐type frequency, a reduction in fibre CSA, and changes in fibre size distribution in *Hmox1/2* ^*−/−*^ compared to *Hmox1/2* ^*+/−*^ and *Hmox1* ^*fl/fl*^ controls (Figure 2b,c). These changes resembled those in *MHmox1* ^*−/−*^ mice, indicating that HO‐1 is essential for myocyte integrity and function. We measured expressions of MyoD and myogenin, finding higher MyoD levels but 42% lower myogenin in *Hmox1/2* ^*−/−*^ compared to controls (Figure 2d). Additionally, we measured muscle atrophy genes Atrogin‐1 and MuRF1, and Myostatin as a negative muscle growth regulator. These genes were significantly upregulated in *Hmox1/2* ^*−/−*^, indicating increased atrophic signalling (Figure 2e).

**FIGURE 3:**
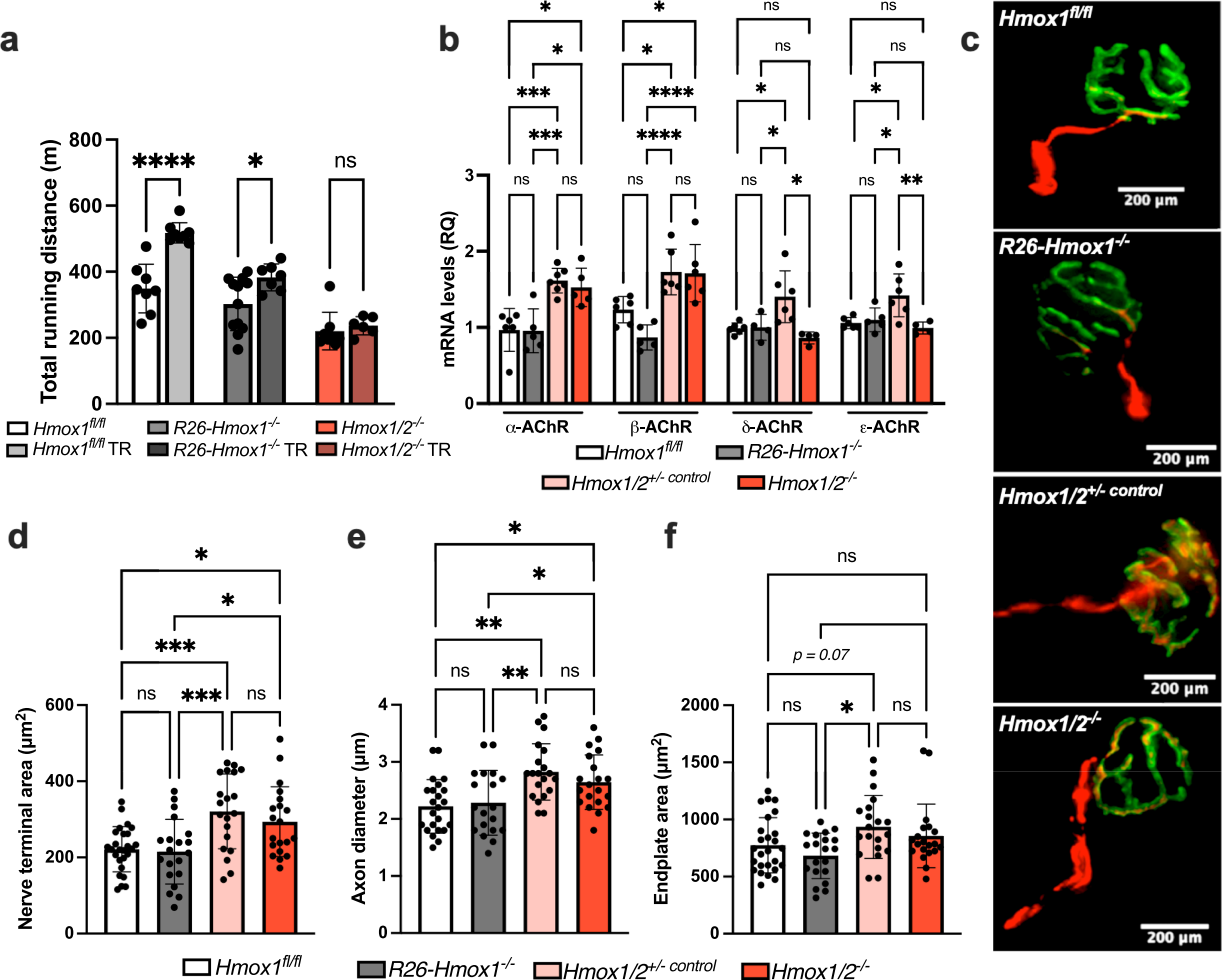
Depletion of HO‐1 and HO‐2 impairs adaptations from aerobic training and affects NMJ morphology (a) Total running distance after 6 weeks of treadmill running training (TR) in *Hmox1* ^*fl/fl*^ mice, global HO‐1 deficient (*R26‐Hmox1* ^*−/−*^) mice, and *Hmox1/2* ^*−/−*^ mice (double HO‐1/HO‐2 knockout). (b) mRNA expression levels for acetylcholine receptor (AChR) subunits in plantaris muscle in *Hmox1* ^*fl/fl*^ mice, global *R26‐Hmox1* ^*−/−*^ mice, *Hmox1/2* ^*+/− control*^ mice (HO‐2 deficient but with normal HO‐1 expression) and *Hmox1/2* ^*−/−*^ mice. (c) Representative immunofluorescent staining images of extensor digitorum longus muscles stained with anti‐*α*‐bungarotoxin (green) and antineurofilament/antisynaptic vesicle (red). Presynaptic measurements of (d) mean area of the nerve terminal, (e) axon diameter and postsynaptic parameters for (f) endplate area as determined using ImageJ software in combination with the BinaryConnectivity plugin. Data are mean ± SD of at least 20 NMJ from four mice/group. *p < 0.05, **p < 0.01, ***p < 0.001 and ****p < 0.0001; *n* = 4–10 mice per group.

The results suggest that HO‐1 primarily maintains the structure, function and homeostasis of muscle fibres. Despite aerobic training, double knockout mice did not improve their running capacity (Figure 3a), contrasting with *Hmox1* ^*fl/fl*^ or *R26‐Hmox1* ^*−/−*^ mice. Performance was more akin to *Hmox2* ^*−/−*^ mice under similar training (Figure 1a). We examined the NMJ in EDL muscle from *Hmox1/2* ^*−/−*^ and noted increased *α*‐ and *β*‐AChR subunits compared to controls (Figure 3b), alongside abnormal NMJ morphology at pre‐ and postsynaptic gaps, influenced by age and mitigated through caloric restriction or exercise [23]. Presynaptic regions in HO‐2‐deficient mice showed enlarged nerve terminal areas and increased axon diameters compared to controls (Figure 3c–e). Similarly, postsynaptic structures in EDL muscle from *Hmox1/2* ^*+/−*^ and *Hmox1/2* ^*−/−*^ mice displayed enlarged endplate areas and increased average AChR cluster sizes (Figure 3f; Figure S2f). These parallel changes suggest that HO‐2, rather than HO‐1, partially regulates neuromuscular synapse architecture. Given these roles and that both heme oxygenases produce bioactive products that regulate cellular function, we investigated whether exogenous CO could substitute for the loss of HO and restore skeletal muscle structure and function.

### 3.3 CO Rescues the Lack of HO and Prevents Skeletal Muscle Abnormalities

Given the critical roles of HO‐1 and HO‐2 in maintaining skeletal muscle integrity and neuromuscular function, we tested whether exogenously delivered CO could counteract the detrimental effects of HO deficiency. HO‐1 depletion in myoblasts (Figure S3a,b) disrupted myogenic processes, leading to dysregulated cell proliferation and increased MyoD expression (Figure S3c), as well as impaired muscle differentiation, evidenced by decreased myogenin and IGF‐1 expression (Figure S3d,e). CO treatment reversed these abnormalities, restoring MyoD, myogenin and IGF‐1 levels to baseline (Figure S3c–e).

**FIGURE 4:**
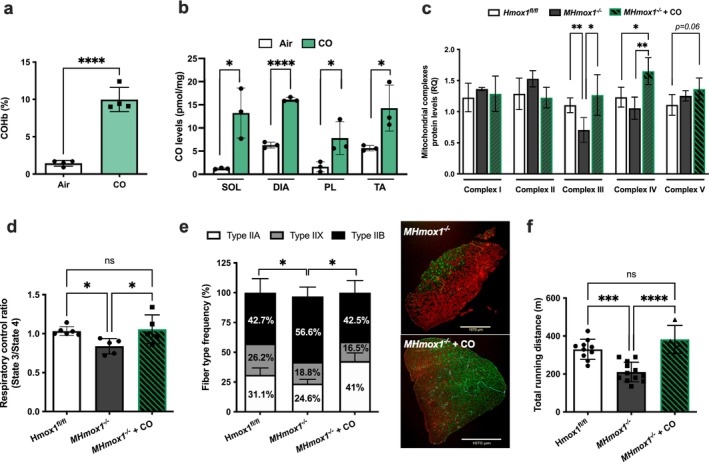
CO prevents muscle abnormalities caused by the lack of HO‐1 (a,b) Carboxyhemoglobin levels and muscle CO concentrations (pmol/mg) were measured after one hour of CO exposure (250 ppm). (c) Quantification of mitochondrial complex expression in plantaris muscle of *Hmox1* ^*fl/fl*^ control and *MHmox1* ^*−/−*^ animals following 14 days of exposure to air or CO (250 ppm; 1 h/day); see also Figure S4d. (d) The respiratory control ratio (State 3ADP/State 4oligomycin) in isolated mitochondria from the gastrocnemius muscle was assessed in the presence of glutamate/malate. (e) Fibre‐type frequency (left) and representative image (right) were isolated from *MHmox1* ^*−/−*^ mice after 14 days of exposure to air or CO. Green: Type IIa MyHC isoform; red: Type IIb MyHC isoform; black: Type IIx MyHC isoform; turquoise: laminin. Data are mean ± SD from six fields at 10x/muscle. *p < 0.05; *n* = 3–5 mice per group. (f) Total running distance on the treadmill. ***p < 0.001 and ****p < 0.0001; *n* = 4–10 mice per group.

**FIGURE 5:**
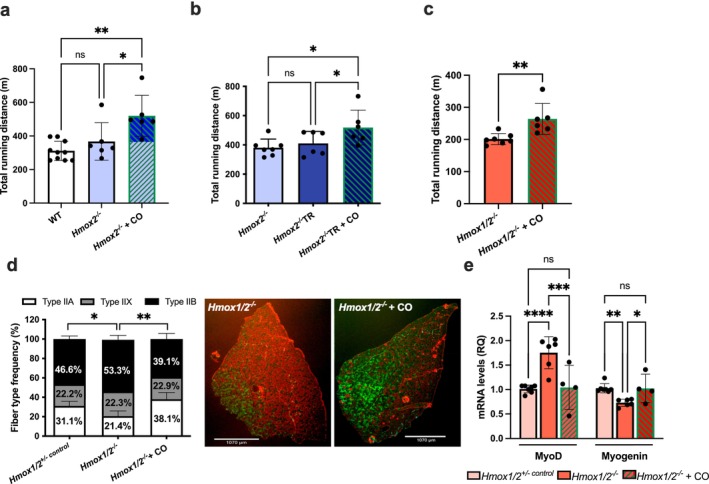
CO restores exercise adaptation and fibre‐type balance in HO‐2 and HO‐1/HO‐2‐deficient mice (a) Total running distance on the treadmill of *Hmox2* ^*−/−*^ animals after exposure to air or CO (250 ppm; 1 h/day) for 14 days, or (b) after 6 weeks of exercise training (TR) and exposure to air or CO (250 ppm; 1 h/day; 5X/week; before each training session). (c) Treadmill total running distance of *HO1/2* ^*−/−*^ mice following 14 days of exposure to air or CO (250 ppm; 1 h/day). (d) Fibre‐type frequency (left) and a representative image (right) from *Hmox1/2* ^*−/−*^ mice after 14 days of exposure to air or CO. Green denotes Type IIa MyHC isoform; red denotes Type IIb MyHC isoform; black denotes Type IIx MyHC isoform; turquoise denotes laminin. Data represent mean ± SD from five fields at 10x/muscle. Scale bars: 1070 μm. *p < 0.05 and **p < 0.01; *n* = 4 mice/group. (e) mRNA expression levels for MyoD and myogenin in the plantaris muscle of *Hmox1/2* ^*+/− control*^ and *Hmox1/2* ^*−/−*^ mice after 14 days of exposure to air or CO (250 ppm; 1 h/day). Data are mean ± SD, **p < 0.01; *n* = 4–7 mice/group.

Exposure to exogenous CO in vivo elevated CO content in the blood (10.3% ± 1.1%) and perfused soleus, diaphragm, plantaris and TA muscle groups (Figure 4a,b). We tested whether CO (1 h/day at 250 ppm for 14 days) would rescue muscle deficiencies in single and double knockouts of HO‐1, HO‐2 and HO‐1/HO‐2. CO treatment restored mitochondrial oxidase Complexes III and IV and mitochondrial fusion and fission levels (Figure 4c; Figure S4a). Our previous work indicated metabolic deficits in mitochondria from HO‐1‐deficient skeletal muscle, showing impaired respiratory rates [9]. CO exposure rescued these deficits to near normal levels (Figure 4d). CO treatment also corrected the abnormal fibre composition in *MHmox1* ^*−/−*^ mice (Figure 4e), normalizing the percentage of muscle fibre types and improving motor function (Figure S4b) and running speed (increasing from 20.7 to 26.3 m/min, Figure S4c), coupled with an 82% increase in running distance (210.5 vs. 382.5 m, *p* < 0.001; Figure 4f). These findings demonstrate that HO‐1‐derived CO modulates mitochondrial function and reverses performance deficits due to HO‐1 deficiency. We next tested whether CO could mitigate altered running performance in the absence of HO‐2 (Figure 1a). Initially, we found that 14 days of daily CO exposure improved the running capacity of *Hmox2* ^*−/−*^ mice at baseline and raised the proportion of oxidative muscle fibre types (Figure 5a; Figure S4e). Next, *Hmox2* ^*−/−*^ mice were treated daily with CO exposure throughout the 6‐week training period (1 h/day; 250 ppm) before each exercise bout. This restored expected gains in aerobic training adaptation otherwise lost without HO‐2 (Figure 5b). Moreover, CO rescued muscle morphology and improved running performance in *Hmox1/2* ^*−/−*^ mice suffering from increased fatigability and inability to adapt to training (Figure 3a; Figure 5c). CO promoted a metabolic shift toward more oxidative, fatigue‐resistant muscle fibre type that countered HO deficiency effects (Figure 5d). This recovery was accompanied by restoring MyoD and myogenin expression to near baseline levels (Figure 5e). Collectively, these results provide evidence that CO compensates for the lack of both HO isoforms, crucial for appropriate skeletal muscle function and exercise performance.

### 3.4 CO Restores Metabolic Balance in HO1/2‐Deficient Skeletal Muscle

**FIGURE 6:**
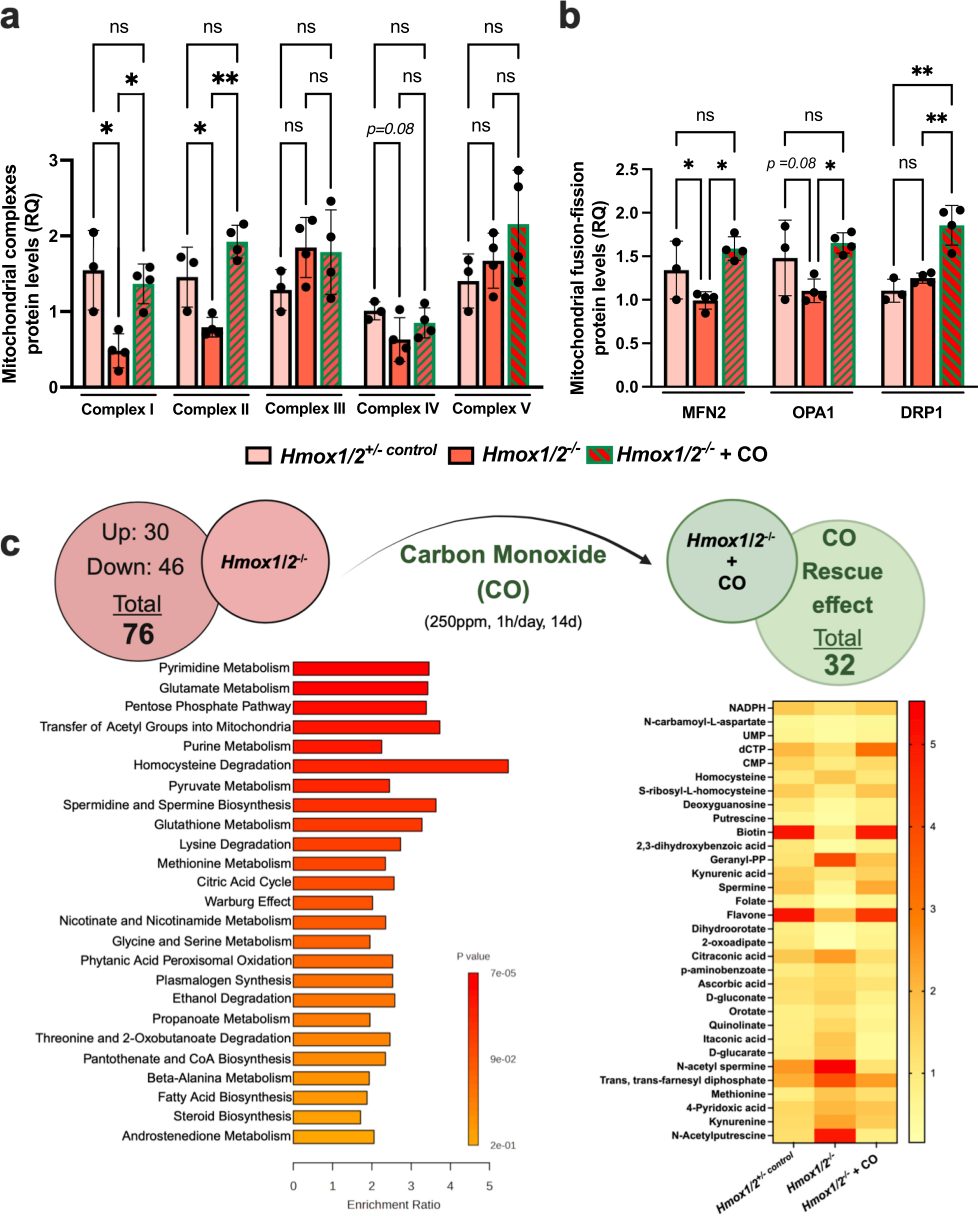
CO rescues metabolic imbalances promoted by HO‐1 and HO‐2 deficiency in skeletal muscle. (a,b) Protein expression levels of mitochondrial complexes and mitochondrial fusion‐fission regulators in plantaris muscle from *Hmox1* ^*fl/fl*^, *Hmox1/2* ^*+/−control*^ and *Hmox1/2* ^*−/−*^ mice exposed to air or CO (250 ppm; 1 h/day for 14 days; see also Figure S6). Data are mean ± SD *p < 0.05 and ** *p* < 0.01; *n* = 3–4 mice per group. (c) Metabolomic pathway enrichment analysis (left) reveals 76 significantly (> 1.5‐fold change) altered metabolites in *Hmox1/2* ^*−/−*^ skeletal muscle compared to *Hmox1/2* ^*+/−control*^ mice, including pyrimidine metabolism, glutamate metabolism, the pentose phosphate pathway and homocysteine degradation. Quantitative enrichment analysis was performed using a generalized linear model to estimate the Q‐statistic for each metabolite set, which described the correlation between compound concentration profiles and the clinical outcomes. Each horizontal bar represents a metabolic pathway whose abscissa indicates the magnitude of the impact factors. The bar colours represent the *p*‐values of the enrichment analysis. Treatment with CO (250 ppm, 1 h/day for 14 days) in *Hmox1/2* ^*−/−*^ mice partially normalized 32 metabolites (right panel, heat map). Heat map colours represent relative abundance (*z*‐score scale), with red indicating higher levels and yellow indicating lower levels.

CO modulates cellular bioenergetics, enhances oxygen consumption and promotes mitochondrial biogenesis in muscles [4]. To investigate how CO affects metabolism and compensates for HO absence, we exposed *Hmox1/2* ^*−/−*^ or vehicle‐treated *Hmox1/2* ^*+/−*^ control mice to CO (1 h/day at 250 ppm for 14 days) and isolated mitochondria from plantaris muscle. Our measurements showed that mitochondria from *Hmox1/2* ^*−/−*^ plantaris muscles had lower expression of mitochondrial oxidative Complex I and II compared to plantaris muscle mitochondria from *Hmox1/2* ^*+/−*^ controls (Figure 6a). Furthermore, MFN2 and OPA1 GTPases, crucial for mitochondrial fusion, were reduced in *Hmox1/2* ^*−/−*^ muscle, highlighting HO‐1's role in preserving mitochondrial integrity (Figure 6b). Notably, CO treatment restored key protein expression levels to near normal, confirming CO's essential role in maintaining mitochondrial function in skeletal muscle.

CO also influences inflammation, cell death and proliferation, indicating its broader metabolic effects [1]. We analysed TA muscle from *Hmox1/2* ^*−/−*^ mice treated with or without CO for 14 days using liquid chromatography–tandem mass spectrometry to create a polar metabolomic profile. Of the 278 metabolites analysed, 76 were dysregulated in the absence of CO (Figure 6c; Table S2). Pathway enrichment analyses showed these alterations focused on nucleotide metabolism, amino acid homeostasis, mitochondrial function and redox balance. Most disrupted pathways included pyrimidine and purine metabolism, as well as the pentose phosphate pathway, suggesting impairments in nucleotide biosynthesis and mitochondrial function. CO reversed many of these metabolic imbalances, restoring 32 metabolites to control levels, highlighting CO's role in regulating key biochemical pathways (Figure 6c). Notable changes included nucleotide precursors, polyamines and redox‐related metabolites. CO also normalized homocysteine and methionine metabolism essential for methylation and mitochondrial function and increased kynurenic acid, and quinolinate, suggesting involvement in tryptophan metabolism. Overall, CO helps mitigate metabolic imbalances, restore nucleotide homeostasis, reduce oxidative stress and enhance mitochondrial function, indicating its therapeutic potential for metabolic dysfunction related to HO deficiency.

### 3.5 CO Exposure Improves Fatigue Resistance and Running Capacity

**FIGURE 7:**
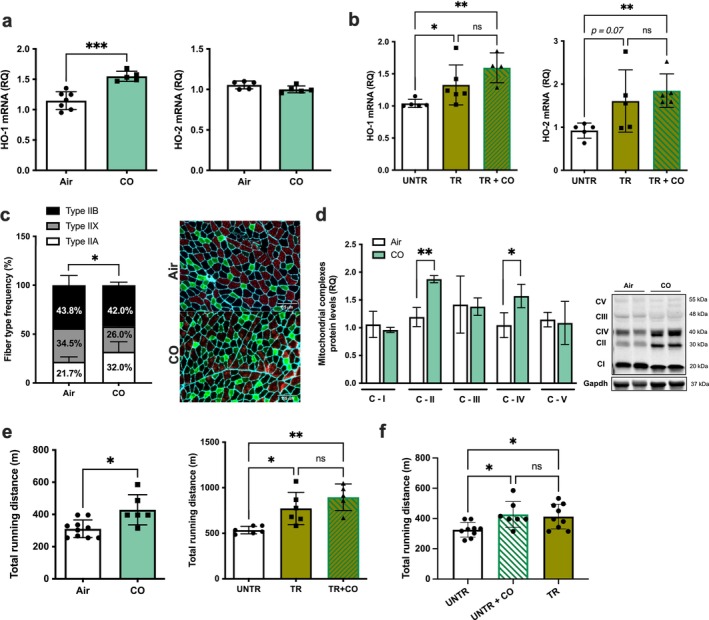
Beneficial effects of CO exposure on skeletal muscle morphology and running capacity (a) The expression levels of HO‐1 and HO‐2 mRNA in the plantaris muscle of animals exposed to air or CO (250 ppm; 1 h/day) for 14 days and (b) before and after 6 weeks of aerobic exercise training (TR) in animals exposed to air or CO (250 ppm; 1 h/day; 5X/week; before each training session). mRNA expression was determined using quantitative RT‐qPCR and normalized to Hprt1. (c) Representative immunofluorescent staining images of tibialis anterior muscle cross sections for muscle fibre‐type frequency. Green, Type IIa MyHC isoform; red, Type IIb MyHC isoform; black, Type IIx MyHC isoform; turquoise, laminin. Images are representative of 10‐week‐old male mice (*n* = 5/group). (d) Quantification of mitochondrial complex expression in the plantaris muscle and a representative immunoblot (*n* = 4/group). (e) Treadmill total running distance measured on Day 14 in WT animals exposed to Air or CO (250 ppm; 1 h/day for 14 days) or before and after 6 weeks of exercise training (250 ppm; 1 h/day; 5X/week; before each training session). (f) Treadmill total running distance. Data are mean ± SD, *p < 0.05; **p < 0.01; ***p < 0.001; *n* = 4–10 mice per group.

Given CO's ability to modulate skeletal muscle physiology, we examined its effect on aerobic capacity. High aerobic capacity, linked to musculoskeletal health and longevity, is a hallmark of HO‐1 and CO activity [9]. We have shown that CO mimics HO‐1's beneficial effects. In plantaris muscle, 2 weeks of daily CO treatment without exercise selectively upregulated HO‐1, whereas HO‐2 levels remained unchanged (Figure 7a; Figure S5a,b). In contrast, 6 weeks of exercise training with or without CO upregulated both HO‐1 and HO‐2 (Figure 7b). CO treatment during training further increased HO‐1 and HO‐2 expression by 19% and 16%, respectively, supporting their role in muscle adaptation.

Skeletal muscle consists of slow and fast‐twitch fibres, affecting fatigue and endurance. CO treatment altered fibre composition in the tibialis anterior muscle, which is similar in composition to plantaris muscle, with an increase by nearly 10% (Figure 7c). Type IIA fibres exhibit lower fatigability and higher oxidative capacity. CO also elevated mitochondrial oxidase Complexes II and IV in plantaris muscle compared to controls (Figure 7d).

Increased HO‐1 in muscle correlates with better exercise performance. We evaluated the effect of CO on running capacity over both short and extended periods. After 2 weeks, male and female mice showed significant improvements in running capacity without changes in haematological parameters (Figure 7e; Figure S5c,d; Table S3). Similarly, mice receiving CO throughout the 6 weeks of training showed a 14% improvement in running performance compared to trained controls, suggesting that CO enhances performance gains (Figure 7e). This was supported by increased oxygen consumption (VO_2_), carbon dioxide production (VCO_2_) and voluntary activity (Figure S5e). Moreover, CO exposure in untrained mice elicited muscular adaptations similar to aerobic training (Figure 7f). These results suggest that skeletal muscle HO produces CO, enhancing muscle function and endurance, and that CO can mimic aerobic exercise as a therapeutic strategy for individuals with mobility challenges.

## 4 Discussion

In our continued effort to understand the biology of heme metabolism and its role in skeletal muscle, our study delineates novel and complementary roles of the heme oxygenase isoforms HO‐1 and HO‐2 in regulating skeletal muscle plasticity, NMJ architecture and the capacity for adaptation to aerobic training. Here, we demonstrate that these two enzymes act synergistically yet nonredundantly to support muscle integrity and function and that their deficiency disrupts homeostasis in both structural and metabolic dimensions. Our findings also reveal that treatment with CO, a by‐product of heme metabolism by HO activity, rescues the physiological deficits caused by HO loss, mimicking the exercise‐induced benefits and suggesting translational therapeutic potential. Collectively, our findings position HO and CO as bioactive signalling molecules involved in regulating skeletal muscle adaptation and supporting overall muscle health.

The HO‐1 and HO‐2 isoforms share similar catalytic mechanisms, substrate specificity and cofactor requirements, with only limited similarities in nucleotide and amino acid sequences [24]. HO‐2 is highly expressed in the brain, where it functions as a crucial regulator of neuronal signalling and homeostasis [25, 26] and is also localized in the postsynaptic membranes of NMJs, suggesting a role in regulating skeletal muscle function [17, 20]. Our findings reveal a complex role for HO‐2 in exercise adaptation and neuromuscular function. Despite preserved sedentary function, HO‐2‐deficient (*Hmox2* ^*−/−*^ *)* mice failed to improve endurance after aerobic training and showed marked NMJ remodelling, including increased expression of AChR subunits and enlarged pre‐ and postsynaptic structures. These findings resemble pathologies in neurodegenerative diseases, such as amyotrophic lateral sclerosis [27] and myasthenia gravis [28], where altered NMJ plasticity precedes functional decline. Notably, the loss of HO‐2 was associated with an accumulation of systemic heme and possibly reduced CO production, suggesting that HO‐2 has a role in heme clearance under physiological conditions. Importantly, although both *R26‐Hmox1* ^*−/−*^ and *Hmox2* ^*−/−*^ mice exhibited increased circulating heme levels after chronic aerobic training, only the absence of HO‐2 limited performance improvement. This dissociation indicates that impaired heme clearance alone is insufficient to account for defective training adaptation. Therefore, HO‐2 constitutive activity and strategic localization at the NMJ suggest a critical role in maintaining local heme homeostasis and, in turn, as a modulator of synaptic stability and muscle training responsiveness. Building on this observation, we propose that HO‐2 supports activity‐dependent neuromuscular plasticity by regulating calcium signalling and downstream transcriptional programmes through CO‐dependent mechanisms. We speculate that HO‐2‐derived CO functions, in part, as a local neuromodulator that fine‐tunes intracellular calcium‐sensitive signalling pathways, processes known to be essential for coupling synaptic activity to long‐term adaptive remodelling [29]. Disruption of HO‐2 impairs this signalling and alters calcium‐dependent transcriptional programmes that include Sox2 expression, which is regulated by activity‐dependent calcium signalling and is important in neuronal plasticity and maturation [21, 22]. Impaired Sox2‐dependent transcriptional programmes may compromise stabilization of adaptive firing patterns, favouring burst‐dominated activity and inefficient network remodelling as was observed in HO‐2‐deficient neurons. In this context, the preservation of baseline neuromuscular function, but failure of training‐induced adaptation, reflects a selective deficit in plasticity rather than basal excitability, positioning an HO‐2‐CO‐Ca^2+^‐Sox2 axis as a key pathway linking synaptic activity to neuromuscular adaptation [30, 31].

Considering HO‐2‐derived CO signalling is important for converting activity into adaptive neuromuscular plasticity, the simultaneous absence of both HO isoforms is likely to exacerbate defects in synaptic signalling, muscle remodelling, and metabolic adaptation. To elucidate the integrated role of HO enzymes in skeletal muscle and neuromuscular function, we generated the *Hmox1/2* ^*−/−*^ mice, thereby markedly reducing HO‐mediated heme degradation and CO production, particularly at the NMJ. These mice exhibited a 36% reduction in running performance, muscle fibre atrophy and elevated expression of muscle atrophy markers, including Atrogin‐1, MuRF1 and Myostatin, along with a shift toward glycolytic fibre types, features associated with reduced endurance and increased fatigability [18]. This fibre‐type remodelling was reflected at the molecular level by an increase in the expression of MyoD and a decrease in myogenin, transcription factors associated with fast‐ and slow‐twitch muscle fibres, respectively [32]. This dysregulation likely indicates an imbalance between proliferation and differentiation under stress, promoting a fatigable phenotype incapable of appropriate adaptation to training. Concomitantly, *Hmox1/2* ^*−/−*^ mice displayed significant NMJ remodelling, including upregulation of AChR subunits *α* and *β*, enlarged endplate areas and expanded nerve terminal morphology, suggesting compensatory responses to disrupted neuromuscular transmission. These changes were not observed in *Hmox1* ^*fl/fl*^ or *R26‐Hmox1* ^*−/−*^ controls, highlighting the contribution of HO‐2 to presynaptic regulation via CO‐dependent modulation of cyclic GMP and calcium signalling [31]. The increased AChR expression and postsynaptic restructuring may reflect homeostatic adaptations driven by oxidative stress–responsive pathways, such as Nrf2 and NF‐κB [7]. Collectively, these data highlight a division of labour in which HO‐1 preserves muscle fibre integrity and metabolic resilience, while HO‐2 maintains NMJ architecture and supports adaptive neuromuscular remodelling in response to physiological stress. The unique remodelling observed in *Hmox1/2* ^*−/−*^ mice illustrates the complex interplay between local heme metabolism, synaptic signalling, and systemic muscle function.

Previous studies have shown that low‐dose CO exposure can modulate inflammation, oxidative stress and mitochondrial function [1, 4]. This intervention effectively reversed metabolic and functional deficits across various models of HO deficiency, including muscle‐specific HO deficiency (*MHmox1* ^*−/−*^), *Hmox2* ^*−/−,*^ and *Hmox1/2* ^*−/−*^ mice. In muscle cells lacking HO‐1, CO treatment normalized the levels of MRFs and muscle growth signals, restoring the balance between proliferation and differentiation processes that were disrupted by satellite cell dysfunction. The beneficial effects of CO also included fibre‐type remodelling, as it increased the proportion of Type IIA oxidative fibres in the tibialis anterior muscle, leading to improved fatigue resistance and fibre composition comparable to control animals. These adaptations in fibre type were associated with enhanced running capacity in all HO‐deficient models and the ability to regain training‐induced improvements in *Hmox2* ^*−/−*^ mice. Furthermore, CO has demonstrated a capacity to modulate presynaptic calcium dynamics and acetylcholine release, suggesting its role in rescuing NMJ function, which may contribute to restoring exercise adaptations in the absence of HO‐2 [29, 31]. Although exogenous CO administration effectively rescues many of the functional, metabolic and neuromuscular consequences of HO deficiency, CO is not able to remove or degrade excess heme. Accumulation of heme is well accepted as a molecule that imparts deleterious effects over time through its pro‐oxidant properties, membrane destabilization and activation of inflammatory signalling pathways, including TLR4‐dependent responses [33]. In this context, CO should be viewed as a downstream signalling product of HO‐1/2 activity, capable of attenuating the toxic consequences of heme accumulation by limiting oxidative stress, suppressing inflammation and promoting mitochondrial biogenesis and bioenergetic efficiency, rather than as a replacement for heme degradation itself. Thus, although CO can restore adaptive responses to exercise, persistent heme accumulation in the absence of its metabolism by HO activity may still predispose tissues to long‐term dysfunction and an inability to adapt, underscoring the importance of heme clearance for sustained tissue homeostasis.

Given that neuromuscular performance relies on synaptic efficiency and muscle metabolism, we explored how the absence of both HO‐1 and HO‐2 impacts mitochondrial bioenergetics. Mice lacking both Hmox1 and Hmox2 exhibited compromised mitochondrial integrity and a reduction in oxidative Complexes I and II, which hindered oxidative fibre function and prompted a shift toward glycolysis. CO countered these deficits by stabilizing mitochondrial dynamics and preserving oxidative metabolism [34]. Metabolomic profiling of the TA muscle revealed significant dysregulation in pathways associated with redox homeostasis, nucleotide biosynthesis and amino acid metabolism, which CO treatment substantially corrected. Specifically, CO restored 32 essential metabolites, including NADPH, polyamines and intermediates from the kynurenine pathway, indicating improvements in redox buffering, methylation capacity and anti‐inflammatory signalling [6, 35]. These results underscore the role of CO as a crucial metabolic modulator functioning downstream of both HO isoforms and suggest that targeted CO therapy could restore muscle energetics in the context of metabolic and structural compromise.

Aerobic capacity is a key determinant of musculoskeletal health, metabolic efficiency and cardiovascular protection, hallmarks commonly associated with HO activity [9, 36]. CO is now recognized as a beneficial gas at low levels and is currently being tested in multiple clinical trials [37]. In skeletal muscle work, CO has been shown to protect against muscle dystrophy and hindlimb ischemia–reperfusion injury [11, 38]. In our study, short‐term CO exposure improved running capacity in both male and female mice, although more studies are required to systematically address sex‐specific regulation of HO signalling and neuromuscular adaptation. Moreover, chronic CO exposure combined with aerobic training resulted in a 15% increase in running capacity compared with controls. CO, when combined with aerobic exercise training, further increased VO_2_, VCO_2_ and voluntary physical activity, suggesting a synergistic effect of CO and exercise. Additionally, CO improved morphological and metabolic adaptations in skeletal muscle, boosting oxidative capacity in a way similar to endurance training. This level of improvement could be clinically significant for conditions marked by exercise intolerance. From a translational perspective, implementing CO‐based therapies will require careful planning regarding delivery methods. CO can be administered through controlled inhalation, CO prodrugs or oral formulations [4, 13, 39], each with unique advantages in pharmacokinetics and tissue targeting. Patient selection is crucial; those with sarcopenia, neuromuscular junction disorders, mitochondrial dysfunction or limited ability to engage in physical training may benefit most from interventions that otherwise promote activity‐dependent plasticity and metabolic resilience. Importantly, CO may function as a biological amplifier of endogenous adaptive pathways, augmenting exercise‐induced neuromuscular and metabolic remodelling in settings where these processes are otherwise compromised. Taken together, CO produced by the heme oxygenases or given exogenously is critical for skeletal muscle function and adaptability.

This study reveals a previously unrecognized relationship between heme regulation and muscle adaptation, including fibre and NMJ changes. Our findings show a clear division of labour between HO‐1, which maintains muscle fibre integrity and metabolic homeostasis, and HO‐2, which is crucial for NMJ plasticity and exercise adaptation. This distinction is evident through our analysis of knockout models, highlighting the differential roles of these enzymes (Table 1). The therapeutic benefits of CO are particularly intriguing. Short‐term CO exposure improved acute exercise endurance and muscle phenotype without affecting haematological parameters, enhancing aerobic exercise training benefits by upregulating HO‐1 and HO‐2 in skeletal muscle. Notably, CO may substitute for exercise training in mice by promoting overall muscle health. Importantly, the restorative effects of CO on mitochondrial function, myogenesis, and oxidative muscle fibres are lost without HO. These findings have significant implications for therapeutic strategies targeting muscle‐related conditions. Identifying roles for HO‐1 and HO‐2 suggests that targeted interventions could be more effective than broad approaches. Thus, CO is emerging as a promising therapeutic agent for conditions characterized by metabolic and neuromuscular dysfunction, serving as a treatment for metabolic disorders and exercise intolerance‐related issues.

**TABLE 1:** Distinct contributions of HO isoforms to muscle physiology and exercise response.

	Muscle health	Running performance	Training adaptation
**Control**	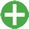	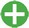	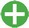
(WT/*Hmox1* ^*fl/fl*^)			
**HO‐1 k.o**.	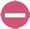	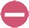	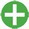
(*MHmox1* ^*−/−*^ /*R26‐cre‐Hmox1* ^*−/−*^)			
**HO‐2 k.o**.	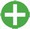	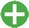	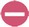
(*Hmox2* ^*−/−*^ /*Hmox1/2+/* ^*−control*^)			
**HO‐1/HO‐2 d.k.o**.	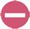	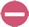	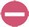
(*Hmox1/2* ^*−/−*^)			

*Note:* 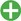: preserved or enhanced function. 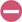: impaired or reduced function relative to control conditions.

## Ethics Statement

The authors certify that they comply with the ethical guidelines for authorship and publishing of the Journal of Cachexia, Sarcopenia and Muscle [40]. All animal studies have been approved by the appropriate ethics committee and have therefore been performed in accordance with the ethical standards laid down in the 1964 Declaration of Helsinki and its later amendments.

## Conflicts of Interest

R.W.A.S. is a scientific advisor for Hillhurst Biopharmaceuticals Inc. L.E.O. is a founder and advisor of GEM‐Biosciences. The remaining authors declare that the research was conducted without commercial or financial relationships that could be construed as a potential conflict of interest. L.E.O. is affiliated with Kyung Hee University, Department of Pharmacology, College of Korean Medicine, Seoul, 02447, Republic of Korea.

## Supporting information


**Figure S1:** The absence of HO‐2 does not alter skeletal muscle morphology or baseline exercise capacity but affects heme metabolism. (a) HO‐2 and HO‐1 mRNA expression in plantaris muscle of WT and *Hmox2* ^*−/−*^ mice. HO‐1 and HO‐2 expression were normalized to Hprt1. (b) Treadmill total running distance for *Hmox2* ^*−/−*^ animals. (c) Representative immunofluorescent staining of tibialis anterior muscles from WT control and *Hmox2* ^*−/−*^ mice, stained with antilaminin (turquoise), anti‐MyHC‐IIa (green) and anti‐MyHC‐IIb (red), fibre‐type frequency measurements from six fields of five animals per group, and mean fibre cross‐sectional area (CSA). Data are mean ± SD *n* = 4–5 per group. (d) mRNA expression in the plantaris muscle of WT control and *Hmox2* ^*−/−*^ animals, normalized to Hprt1. (e) Serum heme levels in WT, global HO‐1 knockout (*R26‐Hmox1* ^*−/−*^) and *Hmox2* ^*−/−*^ animals at baseline and after the end of 6 weeks of aerobic training. (f) Representative spike density maps from individual wells over a 0.5‐s recording window. Each horizontal row represents an active electrode within the well, and vertical lines denote detected spike events. Colour intensity reflects spike density per 1‐ms bin, illustrating increased spike clustering and burst organization in *Hmox2* ^*−/−*^ networks relative to WT. Data are mean ± SD. *p < 0.05, **p < 0.01, ***p < 0.001, ****p < 0.0001; *n* = 4–10 mice per group.
**Figure S2:** Design of HO‐1 and HO‐2 deletion, muscle and NMJ characterization. (a) Breeding strategy to create Heme Oxygenase‐1 (HO‐1, *Hmox1*) and Heme Oxygenase‐2 (HO‐2, *Hmox2*) double knockout. Age‐matched controls consisted of *Hmox1* ^fl/fl^ and *Hmox1/2* ^*+/− control*^ (*olive oil‐Rosa26‐Cre‐Hmox1* ^*+*^ *‐Hmox2* ^*−*^). (b,c) HO‐1 and HO‐2 protein expression with their representative immunoblots in the plantaris muscle. Global deletion of HO‐1 (*R26‐Hmox1* ^*−/−*^) shows similar muscle abnormalities as the muscle‐specific HO‐1 knockdown (*MHmox1* ^*−/−*^) in mice. (d) Tibialis anterior (TA) and plantaris muscle mass normalized to individual body weight. (e) Fibre‐type frequency measurement from six fields of five animals/group. (f) Mean area of AChR clusters as determined using ImageJ software in combination with the BinaryConnectivity plugin. Data are mean ± SD. *p < 0.05, **p < 0.01, ****p < 0.0001; *n* = 6–9 animals per group.
**Figure S3:** C2C12 myoblasts transfected with siRNA duplexes targeting HO‐1 (siHO1) or scrambled control (Scramble) were exposed to an incubator air room or CO (250 ppm) for 72 h. (a) Representative image of C2C12 myoblasts 72 h of siHO‐1 transfection. Note the induction of cell proliferation and the rescue effects of CO. (b,e) mRNA relative quantification (RQ) levels in C2C12 myoblasts after scrambled siRNA (Scramble) or siHO‐1 transfection and air or CO exposure for 72 h. Data are mean ± SD of three independent assays. *p < 0.05, **p < 0.01, ***p < 0.001, ****p < 0.0001; *n* = 3 independent assays.
**Figure S4:** (a) Quantification and western blot membranes of mitochondrial fusion‐fission protein expression in plantaris muscle from *Hmox1* ^*fl/fl*^ control and animals lacking HO‐1 in the skeletal muscle after 14 days of exposure to air room (*MHmox1* ^*−/−*^) or CO (250 ppm, 1 h/day; *MHmox1* ^*−/−*^ + CO). MFN1, MFN2, OPA1 and DRP1 mitochondrial fusion‐fission levels were measured using Cell Signalling antibodies: Mitofusin 1 (#14739), Mitofusin 2 (#9482), OPA1 (#80471) and DRP1 (#5391). GAPDH (Abcam, ab9485) was used as a housekeeping protein. (b) Time on rotarod performance test and (c) maximal running speed on a treadmill exhaustive test. (d) Western blot membranes of mitochondrial Complexes I, II, III, IV and V were measured using a Total OXPHOS Rodent WB antibody cocktail (MitoSciences/Abcam, #MS604/ab110413) in plantaris muscle from *Hmox1* ^*fl/fl*^ control, *MHmox1* ^*−/−*^ or *MHmox1* ^*−/−*^ + CO. (e) Fibre‐type frequency measurement from WT control and animals lacking HO‐2 after 14 days of exposure to air room or CO (250 ppm, 1 h/day; *Hmox2* ^*−/−*^ + CO); six fields of five animals/group. Data are mean ± SD. *p < 0.05, **p < 0.01; *n* = 5 to 10 animals/group.
**Figure S5:** (a,b) Protein expression levels and immunoblots for HO‐1 and HO‐2 in the plantaris muscle of animals exposed to air or CO (250 ppm; 1 h/day) for 14 days. (c,d) Total running distance and maximal running speed in female mice after 14 days of exposure to air room (Air) or carbon monoxide (CO; 250 ppm, ppm; 1 h/day). Data are mean ± SD. *p < 0.05; *n* = 6–8 animals/group. (e) Spontaneous activity level, oxygen consumption (VO_2_), carbon dioxide production (VCO_2_), heat production and respiratory exchange ratio (RER) during the animal's dark phase period (lights off) in animals subjected to 6 weeks of exercise training and exposed to air room or CO (250 ppm, 1 h before each exercise session). Data are mean ± SD. **p < 0.01, ***p < 0.001 vs. TR at same time point, *n* = 5–6 animals/group.
**Figure S6:** Western blot membranes from plantaris muscle of *Hmox1/2* ^*+/− control*^, *Hmox1/2* ^*−/−*^ and *Hmox1/2* ^*−/−*^ + CO animals. (a) Mitochondrial Complexes I, II, III, IV and V were measured using a Total OXPHOS Rodent WB antibody cocktail (MitoSciences/Abcam, #MS604/ab110413). GAPDH (Abcam, ab9485). (b) OPA1, MFN2 and DRP1 mitochondrial fusion‐fission levels were measured using Cell Signalling antibodies: Mitofusin 2 (#9482), OPA1 (#80471) and DRP1 (#5391). GAPDH was used as a housekeeping protein.
**Table S1:** Primer sequences for RT‐qPCR mRNA analysis.
**Table S2:** Metabolites identified in the tibialis anterior muscle of *Hmox1/2* ^*+/− control*^, *Hmox1/2* ^*−/−*^ and *Hmox1/2* ^*−/−*^ animals + CO.
**Table S3:** Haematological measures after 14 days of 1 h daily CO exposure.
