# Supplementary material for: Heme Metabolism‐Derived Carbon Monoxide Regulates Skeletal Muscle Function
Source: J Cachexia Sarcopenia Muscle. 2026 May 14;17(3):e70309. doi: 10.1002/jcsm.70309 (PMC13176642; doi:10.1002/jcsm.70309)
Supplement: Supplementary file 1 — Figure S1: The absence of HO‐2 does not alter skeletal muscle morphology or baseline exercise capacity but affects heme metabolism. (a) HO‐2 and HO‐1 mRNA expression in plantaris muscle of WT and Hmox2 −/− mice. HO‐1 and HO‐2 expression were normalized to Hprt1. (b) Treadmill total running distance for Hmox2 −/− animals. (c) Representative immunofluorescent staining of tibialis anterior muscles from WT control and Hmox2 −/− mice, stained with antilaminin (turquoise), anti‐MyHC‐IIa (green) and anti‐MyHC‐IIb (red), fibre‐type frequency measurements from six fields of five animals per group, and mean fibre cross‐sectional area (CSA). Data are mean ± SD n = 4–5 per group. (d) mRNA expression in the plantaris muscle of WT control and Hmox2 −/− animals, normalized to Hprt1. (e) Serum heme levels in WT, global HO‐1 knockout (R26‐Hmox1 −/− ) and Hmox2 −/− animals at baseline and after the end of 6 weeks of aerobic training. (f) Representative spike density maps from individual wells over a 0.5‐s recording window. Each horizontal row represents an active electrode within the well, and vertical lines denote detected spike events. Colour intensity reflects spike density per 1‐ms bin, illustrating increased spike clustering and burst organization in Hmox2 −/− networks relative to WT. Data are mean ± SD. *p < 0.05, **p < 0.01, ***p < 0.001, ****p < 0.0001; n = 4–10 mice per group. Figure S2: Design of HO‐1 and HO‐2 deletion, muscle and NMJ characterization. (a) Breeding strategy to create Heme Oxygenase‐1 (HO‐1, Hmox1) and Heme Oxygenase‐2 (HO‐2, Hmox2) double knockout. Age‐matched controls consisted of Hmox1 fl/fl and Hmox1/2 +/− control (olive oil‐Rosa26‐Cre‐Hmox1 + ‐Hmox2 − ). (b,c) HO‐1 and HO‐2 protein expression with their representative immunoblots in the plantaris muscle. Global deletion of HO‐1 (R26‐Hmox1 −/− ) shows similar muscle abnormalities as the muscle‐specific HO‐1 knockdown (MHmox1 −/− ) in mice. (d) Tibialis anterior (TA) and plantaris muscle mass normalized t [file JCSM-17-e70309-s001.docx]

**Heme Metabolism-Derived Carbon Monoxide Regulates Skeletal Muscle Function**

Rodrigo W. Alves de Souza^a^, Hyo In Kim^a^, Paula Ketilly Nascimento Alves^a^, Ailma Oliveira da Paixão^b^, Ashlee Rasmussen^a^, Sidharth Shankar^a^, James Harbison^a^, Vanessa Azevedo Voltarelli^a^, Leo E. Otterbein^a,c*^

*Leo Otterbein

Professor, Harvard Medical School

Beth Israel Deaconess Medical Center

Email: [lotterbe@bidmc.harvard.edu](mailto:lotterbe@bidmc.harvard.edu)

**Supplemental Methods**

**Cell Culture**

For cell lines, mouse muscle myoblasts (C2C12; ATCC CRL-1772) were grown in DMEM containing penicillin/streptomycin and 10% fetal bovine serum under standard cell culture conditions. Cells were then transfected (RNAiMAx) with siRNA duplexes targeting HO-1 (s67607) or scrambled control siRNA (100 pmol, Thermo Fisher Scientific). Scrambled control and siHO-1 cells submitted to CO exposure were transferred to an air-tight humidified chamber (C-Chamber; Biospherix Oxycycler) and continuously exposed to 250 parts per million (ppm) CO plus 5% CO_2_ for 72h.

For primary neurons, cells were isolated from the cortex and hippocampus of E16 C57BL/6 (WT) and HO-2 k.o. (*Hmox2^-/-^*) embryos using a papain-based dissociation protocol adapted from Fath et al., 2009 [1]. Briefly, dissected brain tissue was collected in dissection medium containing kynurenic acid to reduce excitotoxicity, enzymatically digested with papain, and sequentially washed with light and heavy trypsin inhibitor solutions before gentle mechanical trituration to obtain a single-cell suspension. Neurons were counted and plated at 80,000 cells/well. Cells were cultured in Neurobasal medium supplemented with B27, GlutaMAX, and L-glutamine on 24-well CytoView Microelectrode Array (MEA) plates (Axion Biosystems) pre-coated with poly-D-lysine and laminin. Cultures were maintained under standard incubator conditions and allowed to mature until day 14, when stable spontaneous network activity was established. Extracellular activity was recorded using the Maestro MEA system at 0h, 24h, and 48h, for 5 min at each time point. Raw signals were analyzed using AxIS Navigator and AxIS Metric Plotting Tool. Spikes were detected using an adaptive threshold, and only active electrodes were included. Primary outcome measures were Weighted Mean Firing Rate (Hz) and Burst Percentage (%), with analysis parameters held constant across genotypes and time points. Multiple wells per genotype were analyzed, with at least 3 independent neuronal preparations.

For in vivo animal studies, isogenic male C57BL/6 mice (wild type, WT) were purchased from Charles River or Jackson Laboratory (25-30g). ﻿Heme oxygenase-2 knockout mice (*Hmox2^−/−^*) were kindly provided by Sylvain Doré (University of Florida, Gainesville, FL). To selectively delete the Hmox1 gene globally, floxed Hmox1 mice (*Hmox1^fl/fl^*) were crossed with mice expressing Cre recombinase under the estrogen receptor T2 (Rosa 26; R26-Cre; Jackson Laboratory, Stock# 008463) to produce *R26-Hmox1^fl/fl^* mice. 10- to 12-week-old *R26-Hmox1^fl/fl^* animals were treated with 2.5 mg/day p.o. of tamoxifen, diluted in olive oil (Sigma Aldrich, St. Louis, MO, USA), for 5 consecutive days, followed by a 7-day treatment-free interval to delete Hmox1 and generate *R26-Hmox1^-/-^* mice. To generate HO-1 and HO-2 double knockout mice, *R26-Cre-Hmox1^-/-^* mice were crossed with *Hmox2^-/-^* mice to create *R26-Cre-Hmox1/2^-/-^* mice. Following a similar tamoxifen regimen, 10- to 12-week-old animals exhibited deletion of HO-1 and HO-2, hereafter referred to as *Hmox1/2^-/-^* mice. Age-matched controls consisted of *Hmox1^fl/fl^* mice and olive oil vehicle (*olive oil-Rosa26-Cre-Hmox1-Hmox2^+/-^*), referred to as *Hmox1/2^+/-control^*, which has a deletion of HO-2 and normal HO-1 expression. Skeletal muscle-specific HO-1 knockout mice (*HSA-MCM-Cre-Hmox1^fl/fl^*; hereafter called *MHmox1^-/-^*) were generated as previously described [2]. To delete skeletal muscle *Hmox 1* in *HSA-MCM-Cre-Hmox1^fl/fl^* mice, 10- to 12-week-old mice were fed a chow containing 400 mg of tamoxifen for one month. The diaphragm, tibialis anterior (TA), soleus, gastrocnemius, and plantaris muscles were carefully harvested and stored at −80°C. Male mice were used for all mechanistic, knockout, and CO-rescue experiments unless otherwise specified. To account for sex differences, female wild-type mice were also used in the CO exposure and exercise performance studies. All mouse procedures were approved by the Beth Israel Deaconess Medical Center (BIDMC) Institutional Animal Care and Use Committee (IACUC, #032-2018; 083-2021) following the Association for the Assessment and Accreditation of Laboratory Animal Care guidelines.

**Treadmill Exercise and Aerobic Training Protocols*.***

All animals were acclimatized to a treadmill (Exer 3/6, Columbus Instruments, USA) over four consecutive days, 10min/day at 6m/min. After acclimatization, animals were subjected to a graded, maximal-exhaustive test. The acute exercise test started at 6 m/min, and the speed was increased by 3 m/min every 3 minutes until mice could not run due to exhaustion [3]. ﻿Tests were carried out by a single observer (RWAS), blinded to the identity of each mouse. Total distance run (meters) and peak workload (meters/min) were recorded. For the running training protocol, mice were subjected to a moderate-intensity aerobic exercise for 60 min, five days/week, for 6 weeks at 60% of maximal workload achieved with a graded treadmill running test as described above, which corresponds with maximal lactate steady state [3]. At the end of the third training week, animals were re-evaluated for running performance to adjust running speed intensity. After six weeks of training protocol, the samples were carefully harvested 72 hours after the last exercise session.

**Mitochondrial Isolation and Activity**

Skeletal muscle mitochondria isolation and function were assessed as previously described [2]. All the procedures followed the manufacturer’s instructions (Agilent SeaHorse, XF cell mito-stress kit). Muscle samples from the gastrocnemius were minced and homogenized in an isolation buffer to release mitochondria from within muscle fibers and later washed in the same buffer in the presence of 1 mg/mL bovine serum albumin. The suspension was homogenized and centrifuged. The mitochondrial pellet was washed, and the final pellet was resuspended in a minimal isolation buffer for function assessment. The experiments with isolated mitochondria (10μg mitochondrial protein/mL) were done in an experimental buffer and were prepared in the presence of malate (Sigma, M1000) and glutamate (Sigma, G1251) substrates (2 mM of each). Oxygen consumption rate (OCR) was measured using an XFp extracellular flux analyzer. Ten micrograms/well of isolated mitochondria in the experimental buffer were seeded in a plate and centrifuged. Each well was filled with assay buffer in the presence of glutamate (5 mM) and malate (5 mM), which were used as substrates. ﻿ADP (4 mM, Sigma 2754) was added to induce state 3 respiratory rates. Addition of oligomycin (2μM) was used to determine state 4 rates. Respiratory control ratios were calculated by the ratio state 3:4. Additionally, 2μM carbonyl ﻿cyanide 4-(trifluoromethoxy) phenylhydrazone (FCCP) ﻿was added to evaluate O_2_ consumption during the mitochondrial uncoupling state. All other procedures followed the manufacturer’s instructions (Agilent SeaHorse, XF cell mito-stress kit).

**Histology**

Fiber frequency and cross-sectional area were measured in the TA muscles after dissection, embedding in OCT, and flash-frozen in liquid nitrogen. ﻿Immunofluorescence was performed on 10 μm muscle cryosections with the following antibodies: anti-myosin IIA (SC-71) and anti-myosin IIB (BF-F3; Developmental Studies Hybridoma Bank). The skeletal muscle cell membrane was stained for laminin (Sigma). Secondary antibodies were coupled to Alexa-488, Alexa-594, or Alexa-647 fluorochromes for immunofluorescence detection. After washing, tissue sections were mounted with Mowiol as previously described [2]. Histological analysis was measured in whole muscle preparations at 10X magnification, and images were captured using a Revolve R4 fluorescence microscopy (Echo app, Echo Laboratories). ﻿Fiber frequency was analyzed using ImageJ software, and muscle fiber cross-sectional area was measured using a freely available and open-source software, SMASH-Semiautomatic Image Processing of Skeletal Muscle Histology [4]. All histological analyses were conducted by a single observer (RWAS).

Neuromuscular junctions (NMJ) were immunohistochemically labeled in extensor digitorum longus (EDL) muscle preparations. The collected muscles were fixed for 3h at 4°C in 4% paraformaldehyde (PFA, ThermoFisher), and then dehydrated in 30% sucrose in 1X phosphate-buffered saline (PBS) at 4°C overnight. The EDL muscle was sectioned in the longitudinal plane at 30 μm. Cryosections were washed in PBS and incubated for 12 h at 4 °C in a blocking solution (Mouse On Mouse, M.O.M. (2 drops/ml), 1% Triton X-100 (Sigma, T8787), and 4% bovine serum albumin - BSA in 1X PBS). After blocking, the sections were incubated at 4°C overnight on an orbital shaker with the primary antibody cocktail consisting of mouse anti-neurofilament (1:50; 2H3; DSHB), mouse anti-synaptic vesicles (1:50; SV2; DSHB), and α-Bungarotoxin-ATTO-488 (1:200; ALO-B-100-AG-0.1; Alomone) for AChRs, in 1% Triton X-100, and 4% BSA in 1X PBS. After washing, the sections were incubated with the corresponding anti-mouse IgG1secondary antibodies Alexa Fluor (AF) 594 (1:200; Invitrogen, A66790) for 4 h at room temperature on an orbital shaker in the dark. Finally, muscle preparations were mounted on glass slides in Vecta Shield (VectorLabs) and stored. At all stages, samples were protected from excessive light exposure before imaging. Fluorescence images and projections of the Z-stack's maximal pixel intensity were acquired at 20X magnification using an Olympus BX62 fitted with a 60x1.42 NA UPlanSApo objective, recorded with an Andor Sona (Oxford Instruments) back-illuminated sCMOS camera controlled by SlideBook 6.2 (Intelligent Imaging Innovations). Pre- and post-synaptic structures at individual NMJs were analyzed using ImageJ software in combination with the BinaryConnectivity plugin, following the protocol described in the NMJ-morph User Guide [5]. ﻿The NMJ architecture characterization was based on the definitions given by Jones et al., 2016 [5]. ﻿NMJs were counted from at least four animals per strain. Quantifications were conducted by a single observer (PKNA) blinded to mouse identity.

**Metabolomics**

Muscle metabolite extraction and preparation were performed as previously described [6]. Briefly, water-soluble metabolites were extracted from 25 mg of tissue with 0.5 ml of ice-cold 80% (v/v) methanol. After grinding the sample on dry ice, the homogenate was incubated for 4h at −80°C, followed by centrifugation at 14,000 x g for 10 min at 4 °C, and the supernatant was saved. A 2^nd^ 0.4 ml of 80 % methanol (−80°C) was added to the pellet, the sample was vortexed, centrifuged at 14,000×g for 10 min at 4°C, and the 2nd supernatant was saved. The two supernatants were combined, dried in a SpeedVac (Savant AS160, Farmingdale, NY), and stored at −80°C until analysis. Each sample was injected into a 5500 QTRAP, using a quantitative polar metabolomics profiling platform with selected reaction monitoring that covers all major metabolic pathways. The platform uses hydrophilic interaction liquid chromatography with positive/negative ion switching to analyze 267 metabolites (289 Q1/Q3 transitions) from a single 15-min targeted liquid chromatography-tandem mass spectrometry (LC-MS/MS) acquisition with a 3-ms dwell time and a 1.55-s duty cycle time. Approximately 10–14 data points were acquired per detected metabolite. Peak areas from the total ion current for each metabolite were integrated using MultiQuant v.3.0 (AB/SCIEX). Metabolite total ion counts for a given transition were normalized to the protein content of matched lysates for each treatment group, and treatment replicates were scaled around their replicate group means to normalize for run order effects between replicate groups [7]. The resultant peak areas were subjected to relative quantitation analyses with MetaboAnalyst 6.0. Further, statistical, enrichment, and pathway impact analyses were performed using MetaboAnalyst 6.0 software.


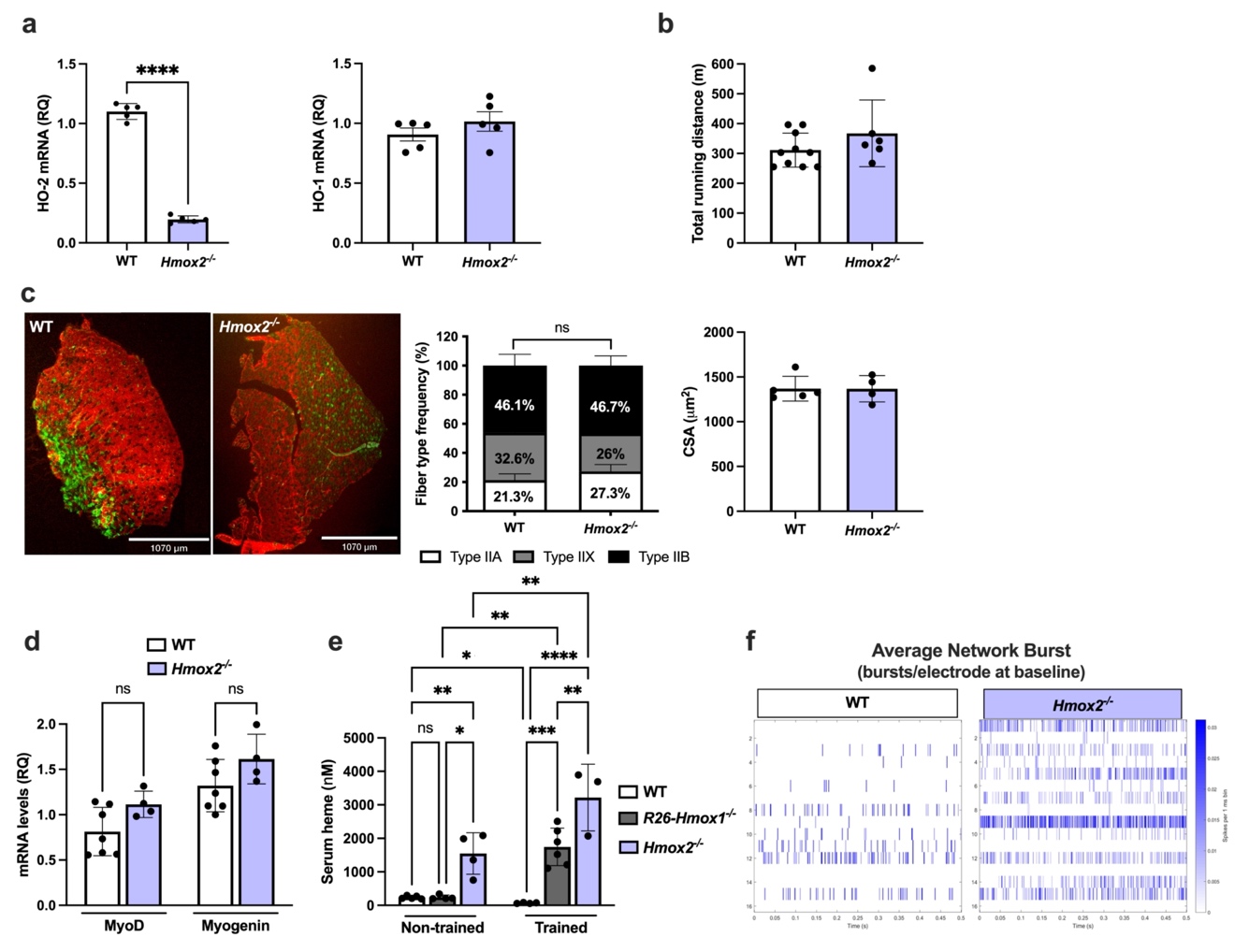


**Fig. S1** The absence of HO-2 does not alter skeletal muscle morphology or baseline exercise capacity but affects heme metabolism. (a) HO-2 and HO-1 mRNA expression in plantaris muscle of WT and *Hmox2^-/-^* mice. HO-1 and HO-2 expression were normalized to Hprt1. (b) ﻿Treadmill total running distance for *Hmox2^-/-^* animals. (c) ﻿Representative immunofluorescent staining of tibialis anterior muscles from WT control and *Hmox2^-/-^* mice, stained with anti-laminin (turquoise), anti-MyHC-IIa (green), and anti-MyHC-IIb (red), fiber-type frequency measurements from six fields of five animals per group, and mean fiber cross-sectional area (CSA). Data are mean ± SD. n = 4-5 per group. (d) mRNA expression in the plantaris muscle of WT control and *Hmox2^-/-^* animals, normalized to Hprt1. (e) ﻿Serum heme levels in WT, global HO-1 knockout (*R26-Hmox1^-/-^*), and *Hmox2^-/-^* animals at baseline and after the end of six weeks of aerobic training. (f) Representative spike density maps from individual wells over a 0.5-s recording window. Each horizontal row represents an active electrode within the well, and vertical lines denote detected spike events. Color intensity reflects spike density per 1-ms bin, illustrating increased spike clustering and burst organization in *Hmox2^-/-^* networks relative to WT. Data are mean ± SD. *p < 0.05, **p < 0.01, ***p<0.001, ****p < 0.0001; n = 4-10 mice per group.


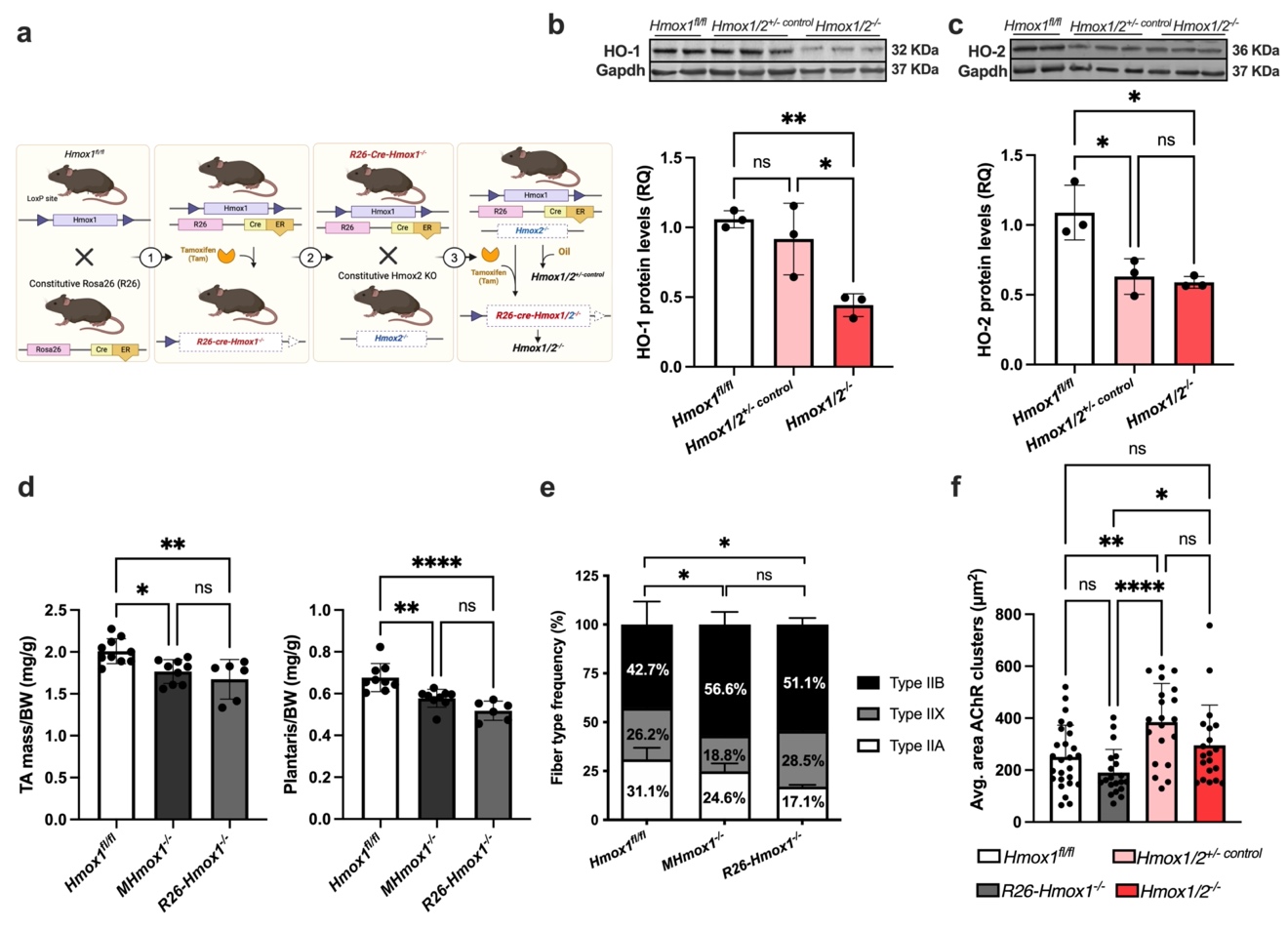
Fig. S2 Design of HO-1 and HO-2 deletion, muscle and NMJ characterization. (a) Breeding strategy to create Heme Oxygenase-1 (HO-1, *Hmox1*) and Heme Oxygenase-2 (HO-2, *Hmox2*) double knock-out. Age-matched controls consisted of *Hmox1*^fl/fl^ and *Hmox1/2^+/- control^* (*olive oil-Rosa26-Cre-Hmox1^+^-Hmox2^-^*). (b-c) HO-1 and HO-2 protein expression with their representative immunoblots in the plantaris muscle. Global deletion of HO-1 (*R26-Hmox1^-/-^*) shows similar muscle abnormalities as the muscle-specific HO-1 knockdown (*MHmox1^-/-^*) in mice. (d) Tibialis anterior (TA) and plantaris muscle mass normalized to individual body weight. (e) Fiber-type frequency measurement from six fields of five animals/group. (f) Mean area of AChR clusters as determined using ImageJ software in combination with the BinaryConnectivity plugin. Data are mean ± SD. *p < 0.05, **p < 0.01, ****p < 0.0001; n=6-9 animals per group.


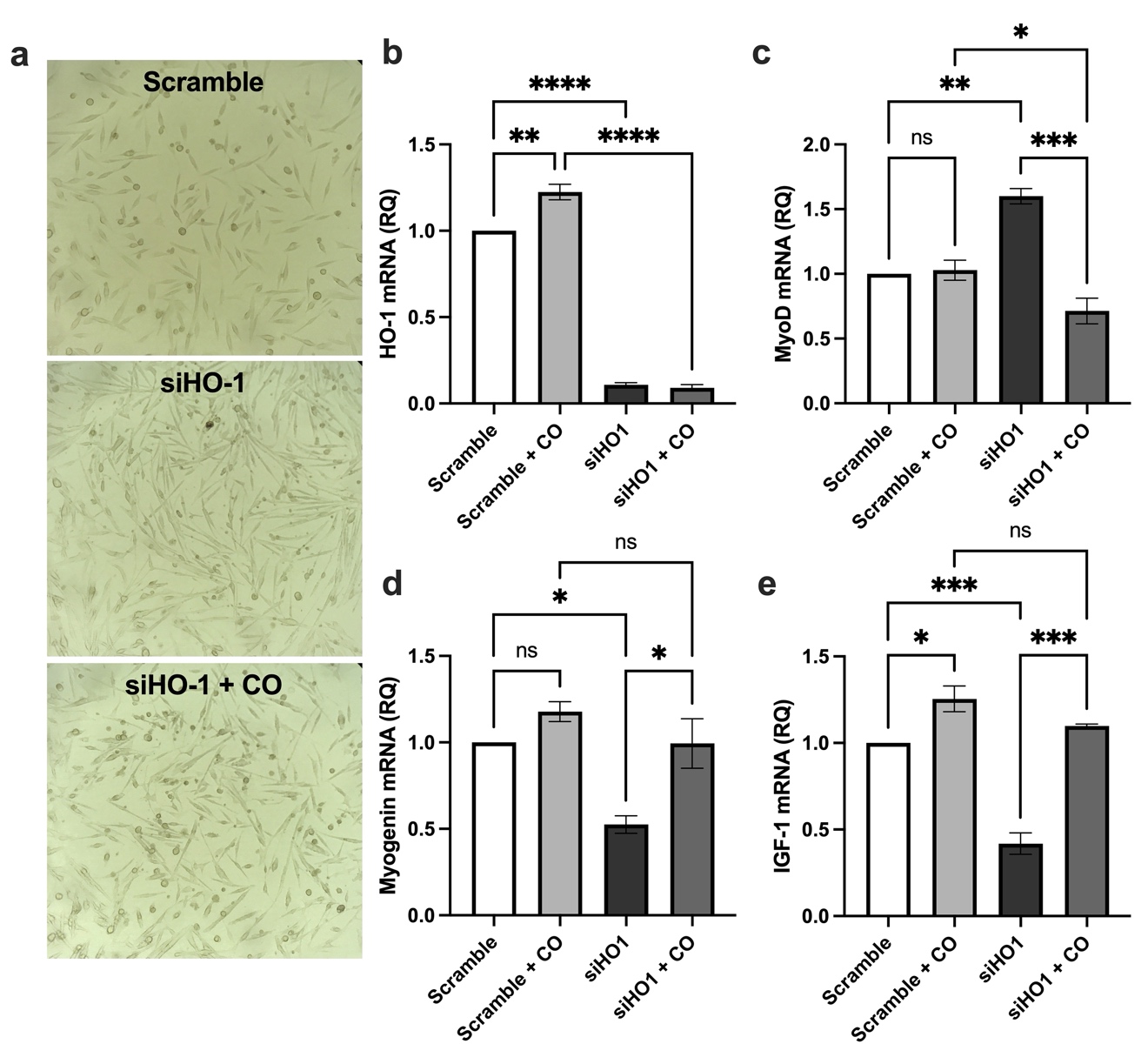


**Fig. S3** C2C12 myoblasts transfected with siRNA duplexes targeting HO-1 (siHO1) or scrambled control (Scramble) were exposed to an incubator air room or CO (250 ppm) for 72h. (a) Representative image of C2C12 myoblasts 72 h of siHO-1 transfection. Note the induction of cell proliferation and the rescue effects of CO. (b-e) mRNA relative quantification (RQ) levels in C2C12 myoblasts after scrambled siRNA (Scramble) or siHO-1 transfection and air or CO exposure for 72h. Data are mean ± SD of three independent assays. *p < 0.05, **p < 0.01, ***p < 0.001, ****p < 0.0001; n=3 independent assays.


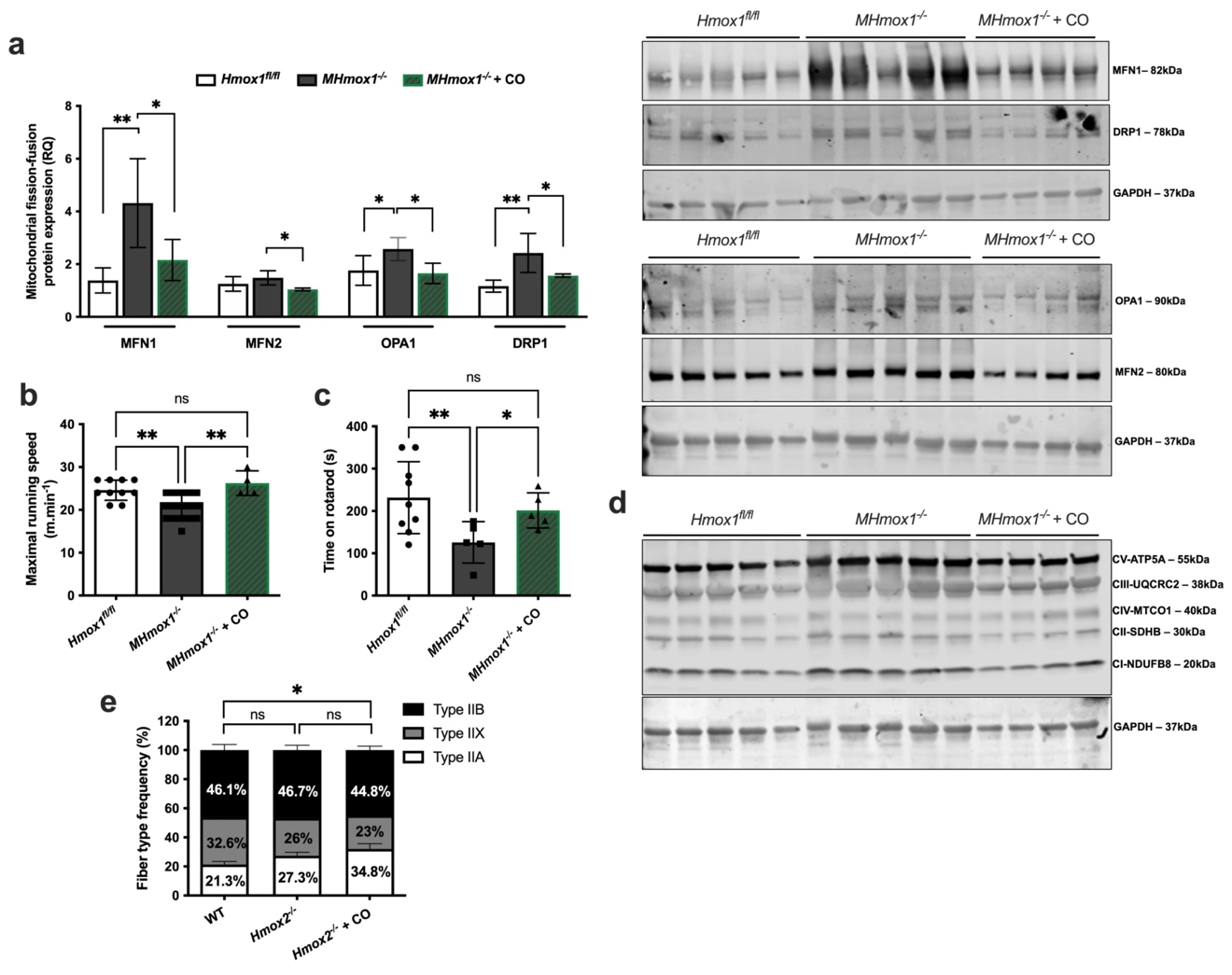
Fig. S4 ﻿﻿(a) ﻿Quantification and western blot membranes of mitochondrial fusion-fission protein expression in plantaris muscle from *Hmox1^fl/fl^* control and animals lacking HO-1 in the skeletal muscle after 14 days of exposure to air room (*MHmox1^-/-^*) or CO (250 ppm, 1h/day; *MHmox1^-/-^* + CO). MFN1, MFN2, OPA1, and DRP1 mitochondrial fusion-fission levels were measured using Cell Signaling antibodies: Mitofusin 1 (#14739), Mitofusin 2 (#9482), OPA1 (#80471), and DRP1 (#5391). GAPDH (Abcam, ab9485) was used as a housekeeping protein. (b) Time on rotarod performance test and (c) maximal running speed on a treadmill exhaustive test. (d) Western blot membranes of mitochondrial complexes I, II, III, IV, and V were measured using a Total OXPHOS Rodent WB antibody cocktail (MitoSciences/Abcam, #MS604/ab110413) in plantaris muscle from *Hmox1^fl/fl^* control, *MHmox1^-/-^* or *MHmox1^-/-^* + CO. (e) Fiber-type frequency measurement from WT control and animals lacking HO-2 after 14 days of exposure to air room or CO (250 ppm, 1h/day; *Hmox2^-/-^* + CO); six fields of five animals/group. Data are mean ± SD. *p < 0.05, **p < 0.01; n=5 to 10 animals/group.


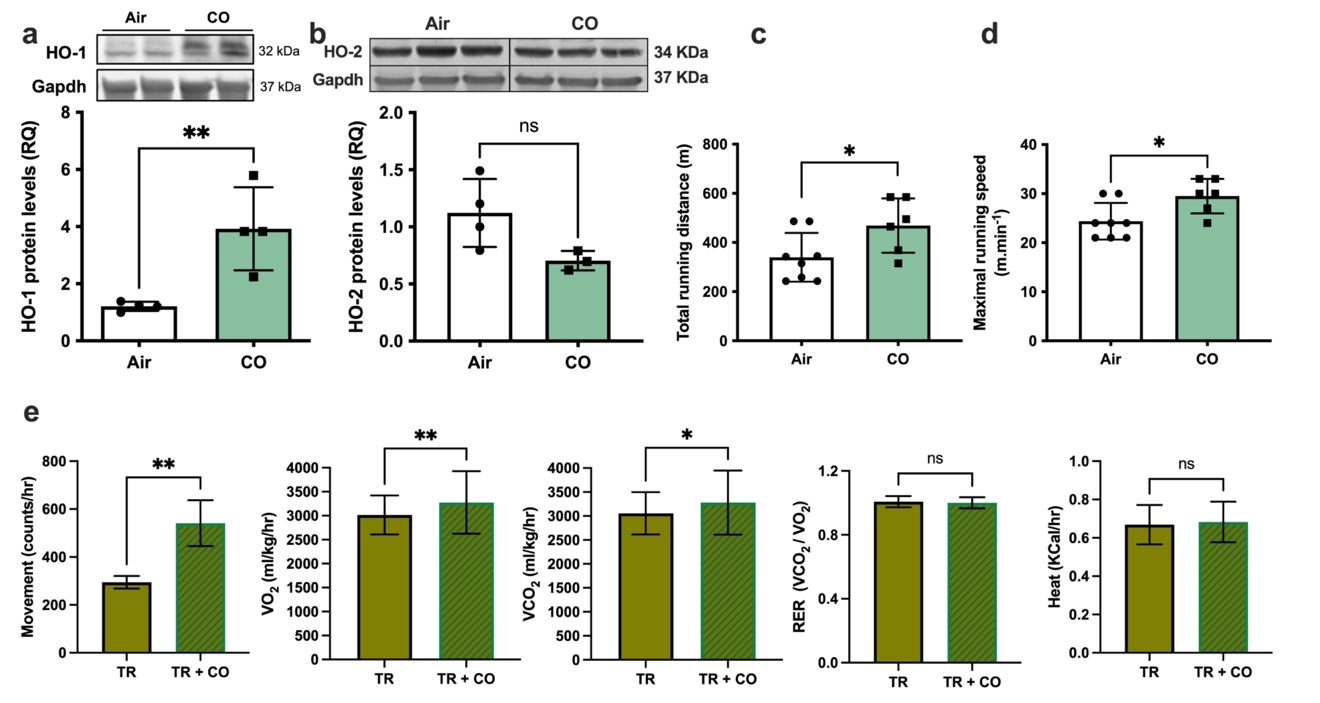


**Fig. S5** (a-b) Protein expression levels and immunoblots for HO-1 and HO-2 in the plantaris muscle of animals exposed to air or CO (250 ppm; 1h/day) for 14 days. (c-d) Total running distance and maximal running speed in female mice after 14 days of exposure to air room (Air) or carbon monoxide (CO; 250 parts per million, ppm; 1h/day). Data are mean ± SD. *p < 0.05; n=6-8 animals/group. (e) Spontaneous activity level, oxygen consumption (VO_2_), carbon dioxide production (VCO_2_), heat production, and respiratory exchange ratio (RER) during the animal’s dark phase period (lights off) in animals subjected to 6 weeks of exercise training and exposed to air-room or CO (250 ppm, 1h before each exercise session). Data are mean ± SD. **p < 0.01, ***p < 0.001 vs TR at same time-point, n=5-6 animals/group.


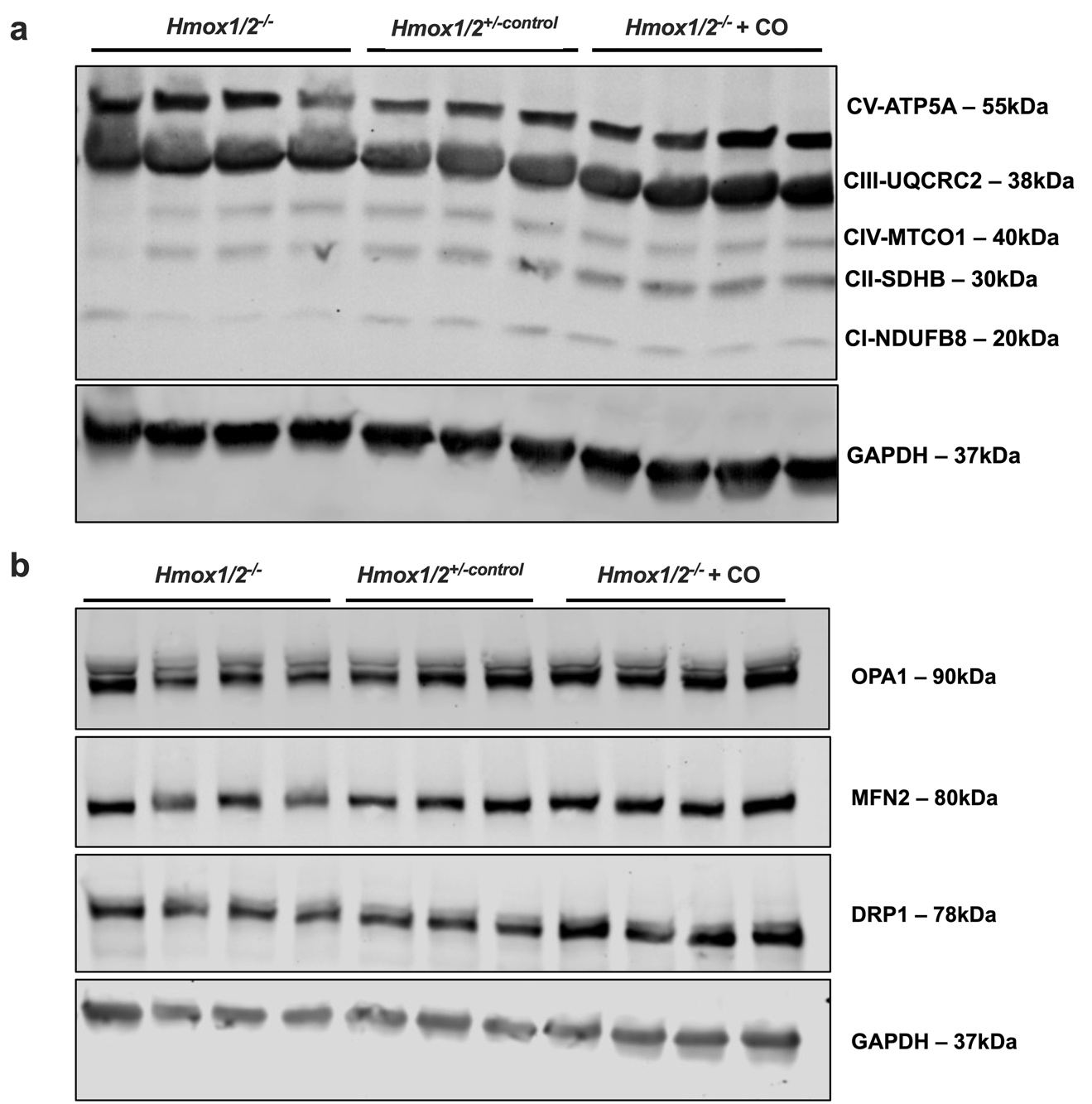


**Fig. S6** Western blot membranes from plantaris muscle of *Hmox1/2^+/- control^*, *Hmox1/2^-/-^* and *Hmox1/2^-/-^* + CO animals. (a) Mitochondrial complexes I, II, III, IV, and V were measured using a Total OXPHOS Rodent WB antibody cocktail (MitoSciences/Abcam, #MS604/ab110413). GAPDH (Abcam, ab9485). (b) OPA1, MFN2, and DRP1 mitochondrial fusion-fission levels were measured using Cell Signaling antibodies: Mitofusin 2 (#9482), OPA1 (#80471), and DRP1 (#5391). GAPDH was used as a housekeeping protein.

Table S1. Primer sequences for RT-qPCR mRNA analysis

| Target gene | PCR Primer sequence 5′ → 3′ | Product Size (bp) | | GenBank Accession # | |  |
| --- | --- | --- | --- | --- | --- | --- |
| Hmox1 | F: CAGAAGAGGCTAAGACCGCC | | 52 | | NM_010442 | |
|  | R: AGCTCCTCAAACAGCTCAATGT | |  |  |  |  |
| Hmox 2 | F: CGGCTGCGAAGGTACAGAGA | | 76 | | NM_01136066.2 | |
|  | R: CTCACTCTGGTCCGTCAGTGG | |  |  |  |  |
| Pax7 | F: GACTCCGGATGTGGAGAAAA | | 145 | | NM_011039 | |
|  | R: GAGCACTCGGCTAATCGAAC | |  |  |  |  |
| MyoD | F: CTGCTCTGATGGCATGATGGA | | 83 | | NM_010866 | |
|  | R: CACTGTAGTAGGCGGTGTCG | |  |  |  |  |
| Myogenin | F: ACTCCCTTACGTCCATCGTG | | 175 | | NM_031189 | |
|  | R: CAGGACAGCCCCACTTAAAA | |  |  |  |  |
| Igf-1 | F: TTACTTCAACAAGCCCACAGG | | 122 | | NM_010512 | |
|  | R: GTGGGGCACAGTACATCTCC | |  |  |  |  |
| Myostatin | F: CTGTAACCTTCCCAGGACCA | | 197 | | NM_010834 | |
|  | R: TCTTTTGGGTGCGATAATCC | |  |  |  |  |
| Atrogin-1 | F: ACGTAGTAAGGCTGTTGGAGC | | 71 | | NM_026346 | |
|  | R: GTTCTTTTGGGCGATGCCAC | |  |  |  |  |
| MuRF1 | F: GAGGGCCATTGACTTTGGGA | | 108 | | NM_001369245 | |
|  | R: TGGTGTTCTTCTTTACCCTCTGT | |  |  |  |  |
| Chrna | F: CCAATAACGCCGCTGAGGAA | | 120 | | NM_007389 | |
|  | R: CAATGAGCCGACCTGCAAAC | |  |  |  |  |
| Chrnb | F: AGCCGAAGGCCAACTGATT | | 140 | | NM_009601 | |
|  | R: CTTCATCCTTCTCGTTCAGGC | |  |  |  |  |
| Chrnd | F: ATCCCCTTGGTAGGCAAGTTC | | 128 | | NM_021600 | |
|  | R: TTGACTCCCTCAGACAGCAC | |  |  |  |  |
| Chrne | F: CAGGAAGCCACTGGAGAGGAAC | | 123 | | NM_009603 | |
|  | R: ACCCCCAAGGAAGATGAGAGT | |  |  |  |  |
| Sox2 | F: GGAGGAGAGCGCCTGTTTTT | | 80 | | NM_011443 | |
|  | R: CTGGCGGAGAATAGTTGGGG | |  |  |  |  |
| Hprt1 | F: CAGTCCCAGCGTCGTGATT | | 138 | | NM_13556 | |
|  | R: GCAAGTCTTTCAGTCCTGTCCAT | |  |  |  |  |
| bp: base pairs | | | | | | |

| Table S2. Metabolites identified in the tibialis anterior muscle of *Hmox1/2^+/-^ ^control^*, *Hmox1/2^-/-^* and *Hmox1/2^-/-^* animals + CO. | | | | |
| --- | --- | --- | --- | --- |
| Metabolite | PubChem ID | | Metabolite | PubChem ID |
| 1-Methyl-Histidine | C01152 | | allantoate | C00499 |
| 1-Methyladenosine | C02494 | | allantoin | C02350 |
| 1,3-diphopshateglycerate | NA | | Aminoadipic acid | C00956 |
| 2-Aminooctanoic acid | NA | | aminoimidazole carboxamide ribonucleotide | C04677 |
| 2-dehydro-D-gluconate | C03342 | | AMP | C00020 |
| 2-Hydroxy-2-methylbutanedioic acid | C00815 | | anthranilate | C00108 |
| 2-hydroxygluterate | C02630 | | arginine | C00062 |
| 2-Isopropylmalic acid | C02504 | | arginosuccinic acid | C03406 |
| 2-keto-isovalerate | C00141 | | Ascorbic acid | C00072 |
| 2-ketohaxanoic acid | NA | | asparagine | C00152 |
| 2-oxo-4-methylthiobutanoate | C01180 | | aspartate | C00049 |
| 2-oxoadipate | C00322 | | ATP-nega | C00002 |
| 2-oxobutanoate | C00109 | | Atrolactic acid | C05584 |
| 2,3-dihydroxybenzoic acid | C00196 | | betaine | C00719 |
| 2,3-Diphosphoglyceric acid | C01159 | | betaine aldehyde | C00576 |
| 3-hydroxybuterate | C01089 | | biotin | C00120 |
| 3-methylphenylacetic acid | NA | | Carbamoyl phosphate | C00169 |
| 3-phospho-serine | C01005 | | carnitine | C00487 |
| 3-phosphoglycerate | C00597 | | CDP-choline | C00307 |
| 4-phosphopantothenate | C03492 | | CDP-ethanolamine | C00570 |
| 4-Pyridoxic acid | C00847 | | CDP-nega | C00112 |
| 5-methoxytryptophan | NA | | Cellobiose | C00185 |
| 5-methyl-THF | NA | | cholesterol | C00187 |
| 5-phosphoribosyl-1-pyrophosphate | C00119 | | cholesteryl sulfate | C18043 |
| 6-phospho-D-gluconate | C00345 | | choline | C00114 |
| 7-methylguanosine | NA | | Citraconic acid | C02226 |
| 7,8-dihydrofolate | NA | | citrate | C00158 |
| a-ketoglutarate | C00026 | | citrate-isocitrate | NA |
| acadesine | NA | | citrulline | C00327 |
| acetoacetate | NA | | CMP | C00055 |
| acetoacetyl-CoA-posi | C00332 | | coenzyme A-posi | NA |
| acetyl-CoA-posi | C00024 | | creatine | C00300 |
| Acetylcarnitine DL | C02571 | | Creatinine | C00791 |
| Acetyllysine | C12989 | | CTP-nega | C00063 |
| acetylphosphate | C00227 | | cyclic-AMP | C00575 |
| aconitate | C00417 | | cystathionine | C02291 |
| adenine | C00147 | | cysteine | C00097 |
| adenosine | C00212 | | cystine | C01420 |
| adenosine 5-phosphosulfate | C00224 | | cytidine | C00475 |
| ADP-D-glucose | C00498 | | cytosine | C00380 |
| ADP-nega | C00008 | | D-erythrose-4-phosphate | C00279 |
| alanine | C01401 | | D-glucarate | C00818 |
| D-gluconate | C00257 | | glutamate | C00302 |
| D-glucono-lactone-6-phosphate | C01236 | | glutamine | C00064 |
| D-glucosamine-1-phosphate | NA | | glutathione | C00051 |
| D-glucosamine-6-phosphate | NA | | glutathione disulfide-nega | NA |
| D-glyceraldehdye-3-phosphate | C00118 | | glutathione disulfide-posi | C00127 |
| D-sedoheptulose-1-7-phosphate | C05382 | | glutathione-nega | C00051 |
| dAMP | C00360 | | Gluterate | C00489 |
| dATP-nega | C00131 | | glycerate | C00258 |
| dCDP-nega | C00705 | | glycerol 3-phosphate | C00093 |
| dCMP | C00239 | | Glycerophosphocholine | C00670 |
| dCTP-nega | C00458 | | glyoxylate | C00048 |
| dehydroascorbic acid | C05422 | | GMP | C00144 |
| deoxyadenosine | C00559 | | GTP-nega | C00044 |
| Deoxycholic acid | C04483 | | Guanidoacetic acid | C00581 |
| deoxyguanosine | C00330 | | guanine | C00242 |
| deoxyinosine | C05512 | | guanosine | C00387 |
| deoxyribose-phosphate | C00673 | | guanosine 5-diphosphate,3-diphosphate | C01228 |
| deoxyuridine | C00526 | | hexose-phosphate | NA |
| dephospho-CoA-nega | C00882 | | histidine | C00135 |
| dephospho-CoA-posi | C00882 | | histidinol | NA |
| dGDP-nega | C00361 | | HMG-CoA_pos | NA |
| dGMP | C00362 | | homocysteine | C00155 |
| dGTP | C00286 | | homoserine | C00263 |
| dihydroorotate | C00337 | | Hydroxyisocaproic acid | NA |
| dihydroxy-acetone-phosphate | C00111 | | Hydroxyphenylacetic acid | C05852 |
| dimethylglycine | C01026 | | hydroxyphenylpyruvate | C01179 |
| DL-Pipecolic acid | C00408 | | hydroxyproline | C01157 |
| dTDP-nega | C00363 | | hypoxanthine | C00262 |
| dTMP-nega | C00364 | | IDP-nega | C00104 |
| dTTP-nega | C00459 | | Imidazoleacetic acid | C02835 |
| dUTP-nega | C00460 | | IMP | C00130 |
| ethanolamine | C00189 | | indole | C00463 |
| FAD | C00016 | | Indole-3-carboxylic acid | C19837 |
| Flavone | C15608 | | Indoleacrylic acid | NA |
| FMN | C00061 | | inosine | C00294 |
| folate | C00504 | | isocitrate | C00311 |
| fructose-1,6-bisphosphate | C00354 | | itaconic acid | C00490 |
| fructose-6-phosphate | C00085 | | Kynurenic acid | C01717 |
| fumarate | C00116 | | Kynurenine | C00328 |
| GDP-nega | C00035 | | L-arginino-succinate | NA |
| Geranyl-PP | C00341 | | lactate | C00186 |
| glucono-lactone | C00198 | | leucine-isoleucine | NA |
| glucosamine | C00329 | | lysine | C00047 |
| glucose-1-phosphate | C00103 | | malate | C00149 |
| glucose-6-phosphate | C00092 | | Maleic acid | C01384 |
| malonyl-CoA-posi | C00083 | | pantothenate | C00864 |
| methionine | C00073 | | phenylalanine | C00079 |
| Methionine sulfoxide | C02989 | | Phenyllactic acid | C01479 |
| Methylcysteine | C22040 | | Phenylpropiolic acid | NA |
| Methylmalonic acid | C02170 | | phosphocreatine | C02305 |
| methylnicotinamide | C02918 | | phosphoenolpyruvate | C00074 |
| methylsuccinate | C08645 | | Phosphorylcholine | C00588 |
| mevalonate_nega | NA | | proline | C00148 |
| myo-inositol | C00137 | | propionyl-CoA-posi | C00100 |
| N-acetyl spermidine | C00612 | | purine | C15587 |
| N-acetyl spermine | C02567 | | putrescine | C00134 |
| N-acetyl-glucosamine | NA | | pyridoxine | C00314 |
| N-acetyl-glucosamine-1-phosphate | C04501 | | Pyroglutamic acid | C01879 |
| N-acetyl-glutamate | C00624 | | Pyrophosphate | C00013 |
| N-acetyl-glutamine | NA | | quinolinate | C03722 |
| N-acetyl-L-alanine | NA | | retinoic acid | C00777 |
| N-Acetyl-L-alanine_neg | NA | | riboflavin | C00255 |
| N-acetyl-L-aspartic acid | C01042 | | ribose-phosphate | C00117 |
| N-acetylaspartylglutamic acid | C12270 | | S-adenosyl-L-homocysteine-nega | C00021 |
| N-Acetylputrescine | C02714 | | S-adenosyl-L-homoCysteine-posi | C00021 |
| N-carbamoyl-L-aspartate | C00438 | | S-adenosyl-L-methioninamine | NA |
| N-carbamoyl-L-aspartate-nega | NA | | S-adenosyl-L-methionine | C00019 |
| N6-Acetyl-L-lysine | C02727 | | S-methyl-5-thioadenosine | C00170 |
| NAD+_nega | NA | | S-ribosyl-L-homocysteine-nega | C03539 |
| NAD+_posi | C00003 | | S-ribosyl-L-homocysteine-posi | C03539 |
| NADH | C00004 | | sarcosine | C00213 |
| NADH-nega | C00004 | | SBP | NA |
| NADP+_nega | C00006 | | serine | C00065 |
| NADP+_posi | C00006 | | sn-glycerol-3-phosphate | C00093 |
| NADPH | C00005 | | sorbitol | C00794 |
| NADPH-nega | C00005 | | spermidine | C00315 |
| Ng,NG-dimethyl-L-arginine | C03626 | | spermine | C00750 |
| nicotinamide | C00153 | | succinate | C00042 |
| Nicotinamide Riboside | C03150 | | succinyl-CoA-posi | C00091 |
| Nicotinamide ribotide | C00455 | | taurine | C00245 |
| nicotinate | C00253 | | Taurodeoxycholic acid | C05463 |
| O-acetyl-L-serine | C00979 | | thiamine | C00378 |
| O8P-O1P | NA | | Thiamine pyrophosphate | C00068 |
| OBP | NA | | thiamine-phosphate | C01081 |
| ornithine | C00077 | | threonine | C00188 |
| orotate | C00295 | | thymidine | C00214 |
| orotidine-5-phosphate | C01103 | | thymine | C00178 |
| oxaloacetate | C00036 | | trans, trans-farnesyl diphosphate | C00448 |
| p-aminobenzoate | C00568 | | trehalose-6-Phosphate | C00689 |
| p-hydroxybenzoate | C00156 | | trehalose-sucrose | NA |
| tryptophan | C00078 | | uracil | C00106 |
| tyrosine | C00082 | | Urea | C00086 |
| UDP-D-glucose | C00029 | | Uric acid | C00366 |
| UDP-D-glucuronate | C00167 | | uridine | C00299 |
| UDP-N-acetyl-glucosamine | C00043 | | UTP-nega | C00075 |
| UDP-nega | C00015 | | valine | C00183 |
| UDP-xylose | C00190 | | xanthine | C00385 |
| UMP | C00105 | | xanthosine | C01762 |
|  |  | | Xanthurenic acid | C02470 |
|  |  |  |  |  |
|  |  |  |  |  |
|  |  |  |  |  |

| Table S3. Hematological measures after fourteen days of 1h daily CO exposure | | |
| --- | --- | --- |
|  | Air | CO |
| ctHb, g/dL | 11.5 ± 0.4 | 11.6 ± 0.2 |
| sO_2_, % | 11.1 ± 4.2 | 8.6 ± 0.5 |
| FO_2_Hb, % | 10.7 ± 4.2 | 7.7 ± 0.6 |
| FMetHb, % | 1.5 ± 0.1 | 1.1 ± 0.8 |
| FHHb, % | 85 ± 3.6 | 80.7 ± 1.1 |
| Hct, % | 35.6 ± 1.0 | 36.1 ± 0.1 |
| ctO_2_, Vol% | 1.9 ± 0.7 | 1.3 ± 0.1 |
| Data represent mean ± SD. ctHb indicates total hemoglobin; sO_2_, oxygen saturation; FO_2_Hb, fractional oxyhemoglobin; FCOHb, fractional carboxyhemoglobin; FMetHb, fractional methemoglobin; FHHb, fractional deoxyhemoglobin; Hct, hematocrit; ctO_2_, total oxygen content. | | |

References

1. Fath, T.; Ke, Y.D.; Gunning, P.; Götz, J.; Ittner, L.M. Primary Support Cultures of Hippocampal and Substantia Nigra Neurons. *Nat. Protoc.* 2009, *4*, 78–85, doi:10.1038/nprot.2008.199.

2. Alves de Souza, R.W.; Gallo, D.; Lee, G.R.; Katsuyama, E.; Schaufler, A.; Weber, J.; Csizmadia, E.; Tsokos, G.C.; Koch, L.G.; Britton, S.L.; et al. Skeletal Muscle Heme Oxygenase-1 Activity Regulates Aerobic Capacity. *Cell Rep.* 2021, *35*, 109018, doi:10.1016/j.celrep.2021.109018.

3. Ferreira, J.C.B.; Rolim, N.P.L.; Bartholomeu, J.B.; Gobatto, C.A.; Kokubun, E.; Brum, P.C. Maximal Lactate Steady State in Running Mice: Effect of Exercise Training. *Clin. Exp. Pharmacol. Physiol.* 2007, *34*, 760–765, doi:10.1111/j.1440-1681.2007.04635.x.

4. Smith, L.R.; Barton, E.R. SMASH - Semi-Automatic Muscle Analysis Using Segmentation of Histology: A MATLAB Application. *Skelet. Muscle* 2014, *4*, 21, doi:10.1186/2044-5040-4-21.

5. Jones, R.A.; Reich, C.D.; Dissanayake, K.N.; Kristmundsdottir, F.; Findlater, G.S.; Ribchester, R.R.; Simmen, M.W.; Gillingwater, T.H. NMJ-Morph Reveals Principal Components of Synaptic Morphology Influencing Structure-Function Relationships at the Neuromuscular Junction. *Open Biol.* 2016, *6*, doi:10.1098/rsob.160240.

6. Yuan, M.; Breitkopf, S.B.; Yang, X.; Asara, J.M. A Positive/Negative Ion-Switching, Targeted Mass Spectrometry-Based Metabolomics Platform for Bodily Fluids, Cells, and Fresh and Fixed Tissue. *Nat. Protoc.* 2012, *7*, 872–881, doi:10.1038/nprot.2012.024.

7. van den Berg, R.A.; Hoefsloot, H.C.J.; Westerhuis, J.A.; Smilde, A.K.; van der Werf, M.J. Centering, Scaling, and Transformations: Improving the Biological Information Content of Metabolomics Data. *BMC Genomics* 2006, *7*, 1–15, doi:10.1186/1471-2164-7-142.
